# Synthesis of 4-Methoxybenzoylhydrazones and Evaluation of Their Antiglycation Activity

**DOI:** 10.3390/molecules19011286

**Published:** 2014-01-21

**Authors:** Muhammad Taha, Humera Naz, Saima Rasheed, Nor Hadiani Ismail, Aqilah Abd Rahman, Sammer Yousuf, Muhammad Iqbal Choudhary

**Affiliations:** 1 Atta-ur-Rahman Institute for Natural Product Discovery, Universiti Teknologi MARA, Puncak Alam Campus, Shah Alam 42300, Malaysia; 2 Faculty of Applied Sciences, Universiti Teknologi MARA, Shah Alam 40450, Malaysia; 3 Faculty of Pharmacy, Universiti Teknologi MARA, Puncak Alam, Shah Alam 42300, Malaysia; 4 H.E.J. Research Institute of Chemistry, International Center for Chemical and Biological Sciences, University of Karachi, Karachi 75270, Pakistan

**Keywords:** 4-methoxybenzoylhydrazones, antiglycation activity, rutin, AGEs, protein glycation inhibition, diabetes

## Abstract

A series of 4-methoxybenzoylhydrazones **1**–**30** was synthesized and the structures of the synthetic derivatives elucidated by spectroscopic methods. The compounds showed a varying degree of antiglycation activity, with IC_50_ values ranging between 216.52 and 748.71 µM, when compared to a rutin standard (IC_50_ = 294.46 ± 1.50 µM). Compounds **1** (IC_50_ = 216.52 ± 4.2 µM), **3** (IC_50_ = 289.58 ± 2.64 µM), **6** (IC_50_ = 227.75 ± 0.53 µM), **7** (IC_50_ = 242.53 ± 6.1) and **11** (IC_50_ = 287.79 ± 1.59) all showed more activity that the standard, and these compounds have the potential to serve as possible leads for drugs to inhibit protein glycation in diabetic patients. A preliminary SAR study was performed.

## 1. Introduction

Benzoylhydrazones have many applications in medicinal and analytical chemistry [1,2,3]. Benzoylhydrazones of different heterocyclic compounds were reported to possess antiproliferative [4], anticonvulsant [5], antioxidant [6], cytotoxicity and anti-HIV activities [7,8]. Numerous benzoylhydrazones have shown interesting bioactivities, such as antibacterial, antifungal, antiinflammatory, antimalarial, analgesic, antiplatelet, anticancer, antituberculosis [9,10,11,12,13,14,15,16,17], insecticidal, antiplasmodium, and antimycobacterial effects, as adriamycin immunoconjugates, proteinase inhibitors and activity against the parasite *Trypanosoma brucei* [18,19,20,21,22]. Their hydrazide derivatives have shown *β*-glucuronidase inhibition activity [23]. In addition, substituted acylhydrazide Schiff bases are reported to have a wide range of bioactivities, including anticancer [24], antitubercular, and anti-inflammatory activities [25]. Hydrazine derivatives also have several commercial applications [26].

Glycation is a non-enzymatic chemical process in which biomolecules (such as proteins, human DNA, and lipids) are damaged by the attachment of reducing sugars (e.g., glucose), finally leading to the formation of highly reactive so-called advanced glycation end products (AGEs). This process has been associated with deleterious health effects. Protein glycation has been implicated in the development of pathologies associated with diabetes and ageing *etc.* [27]. Therefore, the discovery of anti-glycation agents is among the most promising approaches for the management of late diabetic complications. At present only a few glycation inhibitors are known and the requirement of novel glycation inhibitors is still unmet [28]. With the epidemic-like spread of type-2 diabetes, the onsets of late diabetic complications, such as cardiopathy, retinopathy, neuropathy, nephropathy, are on rise. This is largely due to the formation of advanced glycation end products (AGEs) [29,30]. Major efforts have recently been focused on the discovery of new, safe and effective glycation inhibitors [31]. Few molecules are reported to cleave cross-links formed by AGEs, and possibly provide the exciting opportunity of reversing the process of late diabetic complications [32]. It has been discovered that aged garlic extract possess excellent antiglycation potential *in vitro* [33,34]. Aminoguanidine was found to inhibit AGE formation and prevent retinopathy and diabetic vascular complications in diabetic animals, but it showed toxicity problems in phase III clinical trials [35]. Some other molecules (e.g., spermine, spermidine and polyamines) were also reported to have potent anti-glycation potential, similar to those of aminoguanidine and carnosine, but these compounds have to be addressed in future *in vivo* studies [36]. In the search of new, effective and safe antiglycation agents, we have reported several classes of compounds from natural flora, such as cyclopeptide alkaloids from *Ziziphus oxyphylla* Edgw, polyphenolic compounds from *Parmotrema cooperi*, kaempferol-7-*β*-d-glucopyranoside from *Carum petroselinum*, flavanones and flavones from *Iris tenuifolia* and *Otostegia persica* (Burm.) Boiss, respectively [37,38,39,40]. Along with natural compounds we have also reported different classes of synthetic compounds having antiglycation properties in the recent past, such as acylhydrazide [41], benzophenonehydrazone [42], 2,4,6-trichlorophenylhydrazones [43], oxindole derivatives [44], bis-Schiff bases of isatin [45] and metronidazole esters [46]. The work reported here is in continuation of this same systematic study.

## 2. Results and Discussion

### 2.1. Chemistry

**Scheme 1:**
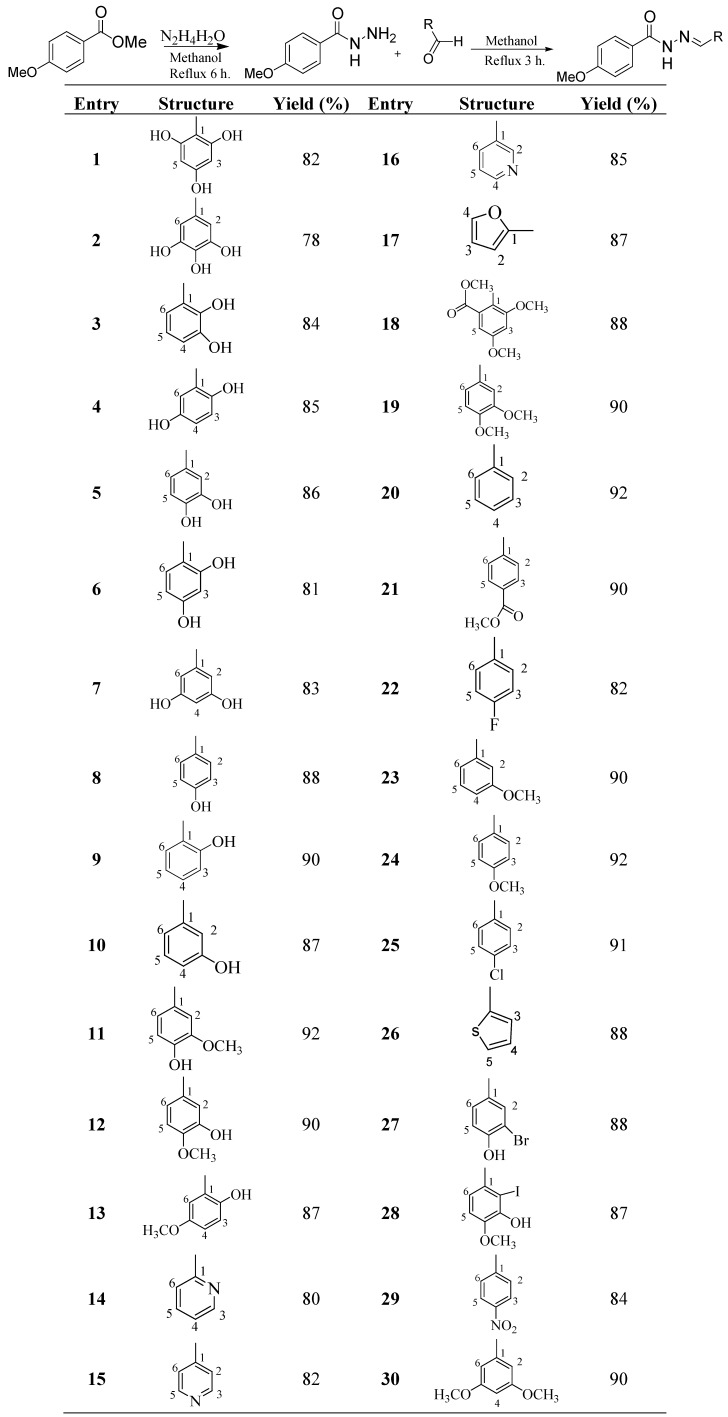
Synthesis of 4-Methoxybenzoylhydrazones **1**–**30**.

4-Methoxybenzoylhydrazones **1**–**30** were synthesized from 4-methoxybenzoylhydrazide, which were obtained from methyl 4-methoxybenzoate by refluxing with hydrazine hydrate for 2 h. The 4-methoxybenzoylhydrazide obtained was recrystallized from methanol. 4-Methoxy- benzoylhydrazones **1**–**30** were prepared by refluxing 4-methoxybenzoylhydrazide with different aldehydes in methanol for 3 to 4 h (Scheme 1). The crude products were further recrystallized from methanol and mostly needle-like crystals were obtained in 78%–92% yield. The structures of the 4-methoxybenzoylhydrazones were deduced using various spectroscopic techniques and CHN analyses. The configuration of C=N double bond is *E*, which can be seen by various crystal structures of similar structures we have published [47,48,49,50,51,52,53,54,55,56].

### 2.2. Antiglycation Activity

#### Structure Activity Relationship

The NH_2_ groups of aminoguanidine and other nitrogen-containing compounds are well known to form Schiff base adducts with the carbonyl moieties of sugars. This interaction is mainly responsible for inhibiting the formation of advanced glycation end product (AGEs). Additionally, it has been found that compounds with different substituents have varying degree of activity against protein glycation [47,48,49,50,51,52,53,54,55,56]. Based on this, we have prepared a series of 4-methoxybenzoylhydrazones **1**–**30** and evaluated their antiglycation potential *in vitro*. For our anti-glycation studies two standards, namely aminoguanidine and rutin, were used. In our protein model system (BSA-MG glycation model), aminoguanidine showed an IC_50_ value of 1168.24 ± 1.2 µM, while rutin showed an IC_50_ value of 294.5 ± 1.5 µM. However, as rutin is more active against glycation than aminoguanidine, we therefore decided to use rutin as the standard in this assay. The compounds **1**–**30** showed potent to moderate antiglycation activities, with IC_50_ values ranging between 216.52 and 748.71 µM, when compared to the standard compound. Compounds **1**, **3**, **6**, **7**, and **11** (IC_50_ = 216.52 ± 4.2 µM, 289.58 ± 2.64 µM, 227.75 ± 0.53 µM, 242.53 ± 6.1 µM and IC_50_ = 287.79 ± 1.59 µM, respectively), showed more potent activities than the rutin standard. The compounds **4** (IC_50_ = 307.1 ± 6.08 µM), **8** (IC_50_ = 347.62 ± 5.8 µM), **2** (IC_50_ = 394.76 ± 3.35 µM) and **12** (IC_50_ = 399.90 ± 7.9 µM) showed good activity. Compounds **5** (IC_50_ = 420.40 ± 3.3 µM) and **17** (IC_50_ = 474.97 ± 19.14 µM) showed moderate activities. Compounds **14** (IC_50_ = 649.18 ± 18.5 µM), **10** (IC_50_ = 657.75 ± 14.0 µM), **18** (IC_50_ = 718.96 ± 10.7 µM) and **15** (IC_50_ = 748.71 ± 7.8 µM) were only weakly active (Table 1).

**Table 1:** *In vitro* protein glycation inhibitory activity of compounds **1**–**30**.

Compounds	IC_50_ (µM ± SEM *^a^*)	Compounds	IC_50_ (µM ± SEM *^a^*)
**1**	216.52 ± 4.2	**16**	NA *^b^*
**2**	394.76 ± 3.35	**17**	474.97 ± 19.14
**3**	289.58 ± 2.64	**18**	718.96 ± 10.7
**4**	307.1 ± 6.08	**19**	NA *^b^*
**5**	420.40 ± 3.3	**20**	NA *^b^*
**6**	227.75 ± 0.53	**21**	NA *^b^*
**7**	242.53 ± 6.1	**22**	NA *^b^*
**8**	347.62 ± 5.8	**23**	NA *^b^*
**9**	NA *^b^*	**24**	NA *^b^*
**10**	657.75 ± 14.0	**25**	NA *^b^*
**11**	287.79 ± 1.59	**26**	NA *^b^*
**12**	399.90 ± 7.9	**27**	NA *^b^*
**13**	NA *^b^*	**28**	NA *^b^*
**14**	649.18 ± 18.5	**29**	NA *^b^*
**15**	748.71 ± 7.8	**30**	NA *^b^*
**Standard Rutin** *^c^*		294.5 ± 1.50	

*^a^* SEM is the standard error of the mean. *^b^* NA Not active. *^c^* Rutin: standard inhibitor for antiglycation activity.

**Figure 1:**
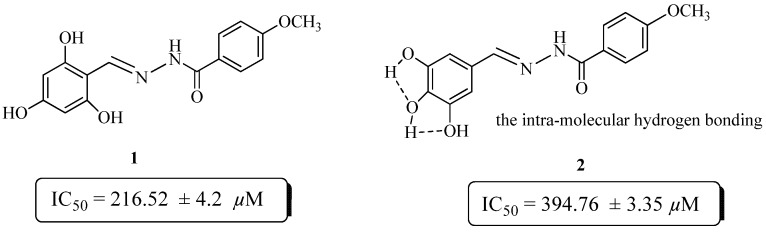
Comparison of the anti-glycation activity of compounds **1** and **2**.

The preliminary structure activity relationship data suggests that the activity mainly depends on the number, as well as the position of hydroxyl substituent’s on the phenyl moiety. Compounds **1** and **2** are both trihydroxy substituted, but compound **1** showed better activity (IC_50_ = 216.52 ± 4.2 µM) than the standard rutin. The activity of these compounds might be due to their capacity to inhibit glycoxidation. Compound **2** showed very low activity (IC_50_ = 394.76 ± 3.35 µM), as compared to compound **1**. This may be due to the intra-molecular hydrogen bonding in compound **2**, which reduce its chances to inhibit glycoxidation as compared to compound **1** (Figure 1).

The five compounds having dihydroxy substituents (*i.e.*, **6**, **7**, **3**, **4** and **5**) showed excellent to moderate activity, depending upon the position of the hydroxyl groups. Compounds **6** (IC_50_ = 227.75 ± 0.53 µM), **7** (IC_50_ = 242.53 ± 6.1 µM) and **3** (IC_50_ = 289.58 ± 2.64 µM) showed more potent activity than the standard (rutin), whereas compounds **4** (IC_50_ = 307.1 ± 6.08 µM) showed activity comparable to the standard. Compound **5** showed moderate activity (Table 1). As discussed earlier, the antiglycation activity mainly depends on the position and potential of hydroxy groups to inhibit glycoxidation. In compounds **6**, the 2,4-dihydroxy groups, being far apart from each other, have no hydrogen bonding with each other. *para*-Hydroxy groups easily inhibit glycoxidation and hence a potent anti-glycation activity was observed. In compound **7**, both hydroxys are at the *meta* position and it showed potent anti-glycation activity, with an IC_50_ value of 242.53 ± 6.1 µM. In compounds **3** and **4**, the *meta*-hydroxy moieties are still free to inhibit glycoxidation, but the activity was decreased with IC_50_ values of 289.58 ± 2.64 and 307.1 ± 6.08 µM, respectively. In compound **5**, the *ortho*-hydroxyl groups are involved in intramolecular hydrogen bonding therefore a weak activity was observed as compared to its analogs, *i.e.*, compounds **6**, **7**, **3** and **4** (Table 1).

The monohydroxyl-substituted analogues showed varied activities, mainly depending on the position of the hydroxyl group. Compound **8** (IC_50_ = 347.62 ± 5.8 µM) is the most active analogue among the monohydroxy derivatives, with a hydroxyl group at the *para* position. When the hydroxy group is at the *meta* position, the activity is reduced by half as compared to compound **8**, (compound **10**; IC_50_ = 657.75 ± 14.0 µM). Interestingly when the hydroxy is at the *ortho* position, as in compound **9**, the activity was completely lost.

Compounds **11**–**13** having one hydroxy and one methoxy group showed varied activity, depending upon the position of the hydroxyl substituent. Compound **11** (IC_50_ = 287.79 ± 1.59 µM) having a *para*-hydroxy, showed better activity than the standard, whereas its analogue **12** (IC_50 _= 399.90 ± 7.9 µM) with a *meta* hydroxy showed a moderate activity against protein glycation. Compound **13** with an *ortho* hydroxy was found to be inactive.

Compounds **14**–**16** possess diverse pyridine rings. The most active among the pyridine derivatives was compound **14** (IC_50_ = 649.18 ± 18.5 µM), with the nitrogen at position-3, near to the hydrazine bridge. The activity decreases sharply when the nitrogen shifts to position-4, as in case of compound **15** (748.71 ± 7.8 µM). Compound **16** with the nitrogen at position-2 was found to be completely inactive (Table 1).

Compounds **17** and **18** showed a weak activity. Compound **17** possess a furfuryl ring and its low activity may be due to the weak interaction of the ring oxygen to inhibit glycoxidation. Furthermore, compound **18** possess an ester moiety, which again interacts weakly with the amino group of the proteins and hence showed a weak activity. Additionally compounds **9**, **13** and **18**–**30** were also found to be inactive.

In conclusion, compounds having hydroxy groups at suitable positions, especially at the *para* position, can inhibit glycoxidation, and thus exhibit a potent antiglycation activity. However, structural modifications can be optimized to achieve the desired activity in this class of compounds.

## 3. Experimental

### 3.1. General Information

NMR experiments were performed on a Bruker Ultra Shield FT NMR 500 MHz (Wissembourg, Switzerland). CHN analysis was performed on a Carlo Erba Strumentazione-Mod-1106 (Milan, Italy). Electron impact mass spectra (EI-MS) were recorded on a Finnigan MAT-311A instrument (Bremen, Germany). Thin layer chromatography (TLC) was performed on pre-coated silica gel aluminum plates (Kieselgel 60, 254, E. Merck, Darmstadt, Germany). Chromatograms were visualized by UV at 254 and 365 nm.

### 3.2. Experimental Protocol

#### 3.2.1. Synthesis of 4-Methoxybenzohydrazide

Methyl 4-methoxybenzoate (10g) was refluxed with the mixture of hydrazine hydrate (10 mL) and methanol (25 mL) for 6 h. The excess hydrazine and methanol were evaporated to give the crude product which was recrystallized from methanol to yield 92% pure 4-methoxybenzohydrazide.

#### 3.2.2. General Procedure for the Synthesis of 4-Methoxybenzohydrazone Derivatives

The 4-methoxybenzohydrazide derivatives were synthesized by refluxing in methanol a mixture of 2 mmol each of 4-methoxybenzohydrazide with different aldehydes and a catalytic amount of acetic acid for 3 h. After the completion of the reaction, the solvent was evaporated under vacuum to afford the crude products which were further recrystallized from methanol to afford needle-like pure products in most of the cases in good to excellent yields.

*N'-(2,4,6-Trihydroxybenzylidiene)-4-methoxybenzohydrazide* (**1**). Solid, M.p.: >250 °C; ^1^H-NMR (DMSO-*d_6_*): δ 11.77 (s, 1H, NH), 11.12 (s, 2H, OH), 9.81 (s, 1H, OH), 8.80 (s, 1H, N=CH-Ar), 7.93 (d, 2H, *J*_2,6/3,5_ = 9.0 Hz, H-2, H-6), 7.07 (d, 2H, *J*_3,5/2,6_ = 9.0 Hz, H-3, H-5), 5.85 (s, 2H, H-3, H-5), 3.83 (s, 3H, OCH_3_); Anal. Calcd for C_15_H_14_N_2_O_5_: C = 59.60, H = 4.67, N = 9.27, O = 26.46, Found C = 59.58, H = 4.65, N = 9.24, O = 26.44; EI MS *m/z* (% rel. abund.): 302. (M^+^, 10), 284 (45), 167 (25), 135 (100).

*N'-(3,4,5-Trihydroxybenzylidiene)-4-methoxybenzohydrazide* (**2**). Solid, M.p.: >250 °C; ^1^H-NMR (DMSO-*d_6_*): δ 11.46 (s, 1H, NH), 11.32 (s, 2H, OH), 9.61 (s, 1H, OH), 8.16 (s, 1H, N=CH-Ar), 7.89 (d, 2H, *J*_2,6/3,5_ = 9.0 Hz, H-2, H-6), 7.07 (d, 2H, *J*_3,5/ 2,6_ = 9.0 Hz, H-3, H-5), 6.70 (s, 2H, H-2, H-6), 3.83 (s, 3H, OCH_3_); Anal. Calcd for C_15_H_14_N_2_O_5_: C = 59.60, H = 4.67, N = 9.27, O = 26.46, Found C = 59.57, H = 4.64, N = 9.25, O = 26.43; EI MS *m/z* (% rel. abund.): 302 (M^+^, 5), 284 (25), 139 (20), 135 (100).

*N'-(2,3-Dihydroxybenzylidene)-4-methoxybenzohydrazide* (**3**). Solid, M.p.: 231°C; ^1^H-NMR (DMSO-*d_6_*): δ 12.01 (s, 1H, NH), 11.26 (s, 1H, OH), 9.61 (s, 1H, OH), 8.58 (s, 1H, N=CH-Ar), 7.95 (d, 2H, *J*_2,6/3,5_ = 9.0 Hz, H-2 , H-6), 7.10 (d, 2H, *J*_3,5/2,6_ = 9.0 Hz, H-3, H-5), 6.96 (dd, 1H, *J*_4,5_ = 6.5, *J*_4,6_ = 2.0 Hz, H-4), 6.86 (dd, 1H, *J*_6,5_ = 6.5, *J*_6,4_ = 2.0 Hz, H-6), 6.76 (t, 1H, *J*_5(4,6)_ = 6.5 Hz, H-5), 3.85 (s, 3H, OCH_3_); Anal. Calcd for C_15_H_14_N_2_O_4_: C = 62.93, H = 4.93, N = 9.79, O = 22.35, Found C = 62.91, H = 4.90, N = 9.77, O = 22.32; EI MS *m/z* (% rel. abund.): 286 (M^+^, 12), 268 (20), 135 (100), 109 (15).

*N'-(2,5-Dihydroxybenzylidene)-4-methoxybenzohydrazide* (**4**). Solid, M.p.: 237 °C; ^1^H-NMR (DMSO-*d_6_*): δ 12.01 (s, 1H, NH), 11.27 (s, 1H, OH), 9.22 (s, 1H, OH), 8.57 (s, 1H, N=CH-Ar), 7.95 (d, 2H, *J*_2,6/ 3,5_ = 9.0 Hz, H-2 , H-6), 7.09 (d, 2H, *J*_3,5/2,6_ = 9.0 Hz, H-3, H-5), 6.96 (dd, 1H, *J*_4,3 _= 8.0, *J*_4,6 _= 2.0 Hz, H-3), 6.86 (d, 1H, *J*_6,4 _= 2.0 Hz, H-6), 6.75 (d, 1H, *J*_3,4_ = 8.0 Hz, H-3), 3.85 (s, 3H, OCH_3_); Anal. Calcd for C_15_H_14_N_2_O_4_: C = 62.93, H = 4.93, N = 9.79, O = 22.35, Found C = 62.91, H = 4.90, N = 9.77, O = 22.31; EI MS *m/z* (% rel. abund.): 286 (M^+^, 6), 268 (18), 135 (100), 109 (18).

*N'-(3,4-Dihydroxybenzylidene)-4-methoxybenzohydrazide* (**5**). Solid, M.p.: 239 °C; ^1^H-NMR (DMSO-*d_6_*): δ 11.47 (s, 1H, NH), 9.41 (s, 2H, OH), δ 8.25 (s, 1H, N=CH-Ar), 7.90 (d, 2H, *J*_2,6/3,5 _= 9.0 Hz, H-2, H-6), (s, 1H, H-6), 7.06 (d, 2H, *J*_3,5/2,6_ = 9.0 Hz, H-3, H-5), 6.93 (d, 1H, *J*_3,2_ = 8.0 Hz, H-4), 6.79 (d, 1H, *J*_2,3_ = 8.0 Hz, H-2), 3.84 (s, 3H, OCH_3_); Anal. Calcd for C_15_H_14_N_2_O_4_, C = 62.93, H = 4.93, N= 9.79, O = 22.35, Found C = 62.91, H = 4.90, N = 9.77, O = 22.32; EI MS *m/z* (% rel. abund.): 286 (M^+^, 17), 268 (22), 135 (100), 109 (9).

*N'-(2,4-Dihydroxybenzylidene)-4-methoxybenzohydrazide* (**6**). Solid, M.p.: >250 °C; ^1^H-NMR (DMSO-*d_6_*): δ 11.85 (s, 1H, OH) 11.56 (s, 1H, OH), 9.98 (s, 1H, OH), 8.41 (s, 1H, N=CH-Ar), 7.92 (d, 2H, *J*_2,6/3,5_ = 9.0 Hz, H-2, H-6), 7.30(d, 1H, *J*_6,5 _= 8.5 Hz, H-6), 7.08 (d, 2H, *J*_3,5/2,6_ = 9.0 Hz, H-3, H-5), 6.37 (dd, 1H, *J*_5,6_ = 8.5, *J*_5,3_ = 2.0 Hz, H-5), 6.32 (d, 1H, *J*_3,5_ = 2.0 Hz, H-3), 3.89 (s, 3H, OCH_3_); Anal. Calcd for C_15_H_14_N_2_O_4_: C = 62.93, H = 4.93, N = 9.79, O = 22.35, Found C = 62.91, H = 4.90, N = 9.77, O = 22.31; EI MS *m/z* (% rel. abund.): 286 (M^+^, 11), 268 (13), 135 (100), 109 (25).

*N'-(3,5-Dihydroxybenzylidene)-4-methoxybenzohydrazide* (**7**). Solid, M.p.: >250 °C; ^1^H-NMR (DMSO-*d_6_*): δ 11.60 (s, 1H, OH) 9.49 (s, 2H, OH), 8.23 (s, 1H, N=CH-Ar), 7.92 (d, 2H, *J*_2,6/3,5_ = 9.0 Hz, H-2, H-6), 7.06 (d, 2H, *J*_3,5/2,6_ = 9.0 Hz, H-3, H-5), 6.60 (s, 2H, H-2, H-6), 6.26 (t, 1H, *J*_4(2,6)_ = 2.0 Hz, H-4), 3.83 (s, 3H, OCH_3_); Anal. Calcd for Anal. Calcd for C_15_H_14_N_2_O_4_: C = 62.93, H = 4.93, N= 9.79, O = 22.35, Found C = 62.91, H = 4.90, N = 9.77, O = 22.31; EI MS *m/z* (% rel. abund.): 286 (M^+^, 6), 268 (17), 135 (100), 109 (22).

*N'-(4-Hydroxybenzylidene)-4-methoxybenzohydrazide* (**8**). Solid, M.p.: >250 °C; ^1^H-NMR (DMSO-*d_6_*): δ 11.54 (s, 1H, NH), 9.93 (s, 1H, OH), 8.32 (s, 1H, N=CH-Ar), 7.90 (d, 2H, *J*_2,6/3,5_ = 9.0 Hz, H-2, H-6), 7.57 (d, 2H, *J*_2,6/3,5_ = 8.5 Hz, H-2/H-6), 7.06 (d, 2H, *J*_3,5/2,6_ = 9.0 Hz, H-3, H-5) 6.84 (d, 2H *J*_3,5/2,6_ = 8.5 Hz, H-3/H-5), 3.83 (s, 3H, OCH_3_); Anal. Calcd for C_15_H_14_N_2_O_3_: C = 66.66, H = 5.22, N= 10.36, O = 17.76, Found C = 66.64, H = 5.20, N = 10.33, O = 17.73; EI MS *m/z* (% rel. abund.): 270 (M^+^, 30), 268 (15), 135 (100), 93 (45).

*N'-(2-Hydroxybenzylidiene)-4-methoxybenzohydrazide* (**9**). Solid, M.p.: 183 °C; ^1^H-NMR (DMSO-*d_6_*): δ 12.02 (s, 1H, NH), 11.40 (s, 1H, OH), 8.62 (s, 1H, N=CH-Ar), 7.95 (d, 2H, *J*_2,6/3,5_ = 9.0 Hz, H-2, H-6), 7.53 (d, 1H, *J*_3,4 _= 7.5, H-3), 7.32 (t, 1H, *J*_5(4,6)_ = 8.5 Hz, H-5), 7.09 (d, 2H, *J*_3,5/2,6_ = 9.0 Hz, H-3, H-5), 6.95–6.90 (m, 2H, H-4/H-6), 3.84 (s, 3H, OCH_3_); Anal. Calcd for C_15_H_14_N_2_O_3_: C = 66.66, H = 5.22, N= 10.36, O = 17.76, Found C = 66.63, H = 5.19, N = 10.32, O = 17.74; EI MS *m/z* (% rel. abund.): 270 (M^+^, 70), 268 (14), 135 (100), 93 (15).

*N'-(3-Hydroxybenzylidene)-4-methoxybenzohydrazide* (**10**). Solid, M.p.: 219 °C; ^1^H-NMR (DMSO-*d_6_*): δ 11.63 (s, 1H, NH), 9.66 (s, 1H, OH), 8.37 (s, 1H, N=CH-Ar), 7.91 (d, 2H, *J*_2,6/3,5_ = 8.5 Hz, H-2, H-6), 7.32 (t, 1H, *J*_5(4,6)_ = 8.5 Hz, H-5), 7.36 (s, 1H, H-2), 7.62 (d, 1H, *J*_6,5 _= 8.0 Hz, H-6), 7.07 (d, 2H, *J*_3,5/2,6_ = 8.5 Hz, H-3, H-5), 6.83 (d, 1H, *J*_4,5_ = 6.5 Hz, H-4), 3.88 (s, 3H, OCH_3_); Anal. Calcd for C_15_H_14_N_2_O_3_: C = 66.66, H = 5.22, N= 10.36, O = 17.76, Found C = 66.63, H = 5.19, N = 10.32, O = 17.74; EI MS *m/z* (% rel. abund.): 270 (M^+^, 87), 268 (15), 135 (100), 93 (25).

*N'-(4-Hydroxy-3-methoxybenzylidene)-4-methoxybenzohydrazide* (**11**). Solid, M.p.: 181.0 °C; ^1^H-NMR (DMSO-*d_6_*): δ 11.57 (s, 1H, NH), 9.56 (s, 1H, OH), 8.33 (s, 1H, N=CH-Ar), 7.91 (d, 2H, *J*_2,6/3,5_ = 8.5 Hz, H-2, H-6), 7.33 (s, 1H, H-2), 7.09 (d, 1H, *J*_6,5_ = 8.0 Hz, H-6), 7.06 (d, 2H, *J*_3,5/2,6_ = 8.5 Hz, H-3, H-5), 6.83 (d, 1H, *J*_5,6_ = 8.0 Hz, H-5), 3.83 (s, 3H, OCH_3_), 3.68 (s, 3H, OCH_3_); Anal. Calcd for C_16_H_16_N_2_O_4_: C = 63.99, H = 5.37, N = 9.33, O = 21.31, Found C = 63.94, H = 5.35, N = 9.31, O = 21.29; EI MS *m/z* (% rel. abund.): 300 (M^+^, 90), 135 (100), 122 (25).

*N'-(3-Hydroxy-4-methoxybenzylidene)-4-methoxybenzohydrazide* (**12**). Solid, M.p.: 213 °C; ^1^H-NMR (DMSO-*d_6_*): δ 11.56 (s, 1H, NH), 9.33 (s, 1H, OH), 8.29 (s, 1H, N=CH-Ar), 7.90 (d, 2H, *J*_2,6/3,5_ = 9.0 Hz, H-2, H-6), 7.27 (s, 1H, H-2), 7.09 (d, 1H, *J*_6,5_ = 8.5 Hz, H-6), 7.06 (d, 2H, *J*_3,5/2,6_ = 9.0 Hz, H-3, H-5), 6.98 (d, 1H, *J*_5,6_ = 8.5 Hz, H-5), 3.84 (s, 3H, OCH_3_), 3.81 (s, 3H, OCH_3_); Anal. Calcd for C_16_H_16_N_2_O_4_: C = 63.99, H = 5.37, N = 9.33, O = 21.31, Found C = 63.94, H = 5.35, N = 9.31, O = 21.29; EI MS *m/z* (% rel. abund.): 300 (M^+^, 70), 135 (100), 122 (30).

*N'-(2-Hydroxy-5-methoxybenzylidene)-4-methoxybenzohydrazide* (**13**). Solid, M.p.: 202 °C; ^1^H-NMR (DMSO-*d_6_*): 11.99 (s, 1H, NH), 10.77 (s, 1H, OH), *δ* 8.60 (s, 1H, N=CH-Ar), 7.94 (d, 2H, *J*_2,6/3,5_ = 8.5 Hz, H-2, H-6), 7.12 (d, 1H, *J*_3,4_ = 8.5 Hz, H-3), 7.09 (d, 2H, *J*_3,5/2,6_ = 8.5 Hz, H-3, H-5), 6.95 (dd, 1H, *J*_4,3 _= 8.5, *J*_4,6_ = 2.0 Hz, H-4), 6.88 (d, 1H, *J*_6,4_ = 2.0 Hz, H-6), 3.84 (s, 3H, OCH_3_), 3.74 (s, 3H, OCH_3_); Anal. Calcd for C_16_H_16_N_2_O_4_: C = 63.99, H = 5.37, N = 9.33, O = 21.31, Found C = 63.97, H = 5.34, N = 9.30, O = 21.28; EI MS *m/z* (% rel. abund.): 300 (M^+^, 90), 135 (100), 122 (21).

*4-Methoxy-N-((pyridine-2-methylene)benzohydrazide* (**14**). Solid, M.p.: 107 °C; ^1^H-NMR (DMSO-*d_6_*): δ 11.96 (s, 1H, NH), 8.62 (d, 1H, *J*_6,5_ = 5.0Hz, H-6), 8.46 (s, 1H, N=CH-Ar), 7.99 (d, 1H, *J*_3,4_ = 8.0 Hz, H-3), 7.94 (d, 2H, *J*_2,6/3,5_ = 8.5 Hz, H-2, H-6), 7.90 (m, 1H, H-4), 7.60 (t, 1H, *J*_5(4,6)_ = 8.0 Hz, H-6), 7.09 (d, 2H, *J*_3,5/2,6_ = 8.5 Hz, H-3, H-5), 3.87 (s, 3H, OCH_3_); Anal. Calcd for C_14_H_13_N_3_O_2_: C = 65.87, H = 5.13, N= 16.46, O = 12.54, Found C = 65.84, H = 5.09, N = 16.44, O = 12.52; EI MS *m/z* (% rel. abund.): 255 (M^+^, 88), 135 (100), 78 (21).

*4-Methoxy-N'-(pyridin-4-methylene)benzohydrazide* (**15**). Solid, M.p.: 180 °C; ^1^H-NMR (DMSO-*d_6_*): δ 12.04 (s, 1H, NH), 8.65 (d, 2H, *J*_2,6/3,5_ = 6.0 Hz, H-2, H-6), 8.42 (s, 1H, N=CH-Ar), 7.93 (d, 2H, *J*_2,6/3,5_ = 8.5 Hz, H-2, H-6), 7.67 (d, 2H, *J*_3,5/2,6_ = 6.0 Hz, H-3, H-5), 7.09 (d, 2H, *J*_3,5/2,6_ = 8.5 Hz, H-3, H-5), 3.84 (s, 3H, OCH_3_); Anal. Calcd for C_14_H_13_N_3_O_2_: C = 65.87, H = 5.13, N= 16.46, O = 12.54, Found C = 65.83, H = 5.11, N = 16.43, O = 12.52; EI MS *m/z* (% rel. abund.): 255 (M^+^, 80), 135 (100), 78 (27).

*4-Methoxy-N'-(pyridin-3-methylene)benzohydrazide* (**16**). Solid, M.p.: 222 °C; ^1^H-NMR (DMSO-*d_6_*): δ 11.92 (s, 1H, NH), 11.92 (s, 1H, H-6), 8.61 (d, 1H, *J*_2,4 _= 2.0 Hz, H-2), 8.36 (s, 1H, N=CH-Ar), 8.16 (d, 1H, *J*_4,5_ = 8.0 Hz, H-4), 7.93 (d, 2H, *J*_2,6/3,5_ = 8.5 Hz, H-2, H-6), 7.51 (dd, 1H, *J*_5,4_ = 8.0 *J*_5,6_ = 5.0 Hz, H-5), 7.08 (d, 2H, *J*_3,5/2,6_ = 8.5 Hz, H-3, H-5), 3.88 (s, 3H, OCH_3_); Anal. Calcd for C_14_H_13_N_3_O_2_: C = 65.87, H = 5.13, N= 16.46, O = 12.54, Found C = 65.82, H = 5.10, N = 16.42, O = 12.52; EI MS *m/z* (% rel. abund.): 255 (M^+^,92), 135 (100), 78 (33).

*N'-((Furan-2-yl)methylene)-4-methoxybenzohydrazide* (**17**). Solid, M.p.: 207 °C; ^1^H-NMR (DMSO-*d*_6_): δ 11.69 (s, 1H, NH), 8.32 (s, 1H, N=CH-Ar), 7.89 (d, 2H, *J*_2,6/3,5_ = 9.0 Hz, H-2, H-6), 7.84 (s, 1H, H-3), 7.07 (d, 2H, *J*_3,5/2,6_ = 9.0 Hz, H-3, H-5), 6.91 (s, 1H, H-3), 6.64 (dd, 1H, *J*_3,4_ = 5.0 Hz, *J*_3,5_ = 2.0 Hz, H-3), 3.83 (s, 3H, OCH_3_); Anal. Calcd for C_13_H_12_N_2_O_3_: C = 63.93, H = 4.95, N= 11.47, O = 19.65, Found C = 63.94, H = 4.97, N = 11.46, O = 19.64; EI MS *m/z* (% rel. abund.): 244 (M^+^, 94), 135 (100), 68 (23).

*Methyl 2-(4-methoxybenzoylimino)methyl)-3,5-dimethoxybenzoate* (**18**). Solid, M.p.: 162 °C; ^1^H-NMR (DMSO-*d_6_*): δ 11.64 (s, 1H, NH), 8.64 (s, 1H, N=CH-Ar), 7.92 (d, 2H, *J*_2,6/3,5_ = 8.5 Hz, H-2, H-6), 7.06 (d, 2H, *J*_3,5/2,6_ = 8.5 Hz, H-3, H-5), 6.76 (d, 1H, *J*_4,6_ = 2.0 Hz, H-4), 6.61 (d, 1H, *J*_6,4_ = 2.0 Hz, H-6), 3.89 (s, 3H, OCH_3_), 3.85 (s, 3H, OCH_3_), 3.84 (s, 3H, OCH_3_); Anal. Calcd for C_19_H_20_N_2_O_6_: C = 61.28, H = 5.41, N= 7.52, O = 25.78, Found C = 61.26, H = 5.43, N = 7.51, O = 25.79; EI MS *m/z* (% rel. abund.): 372 (M^+^, 25), 195 (40), 135 (100).

*N'-(3,4-Dimethoxybenzylidene)-4-methoxybenzohydrazide* (**19**). Solid, M.p.: 179 °C; ^1^H-NMR (DMSO-*d_6_*): δ 11.63 (s, 1H, NH), 8.37 (s, 1H, N=CH-Ar), 7.91 (d, 2H, *J*_2,6/3,5_ = 8.5 Hz, H-2, H-6), 7.36 (s, 1H, H-2), (d, 1H, *J*_6,5 _= 8.5 Hz, H-6), 7.07 (d, 2H, *J*_3,5/2,6_ = 8.5 Hz, H-3, H-5), 6.76 (d, 1H, *J*_5,6_ = 8.5 Hz, H-4), 3.84 (s, 3H, OCH_3_), 3.82 (s, 3H, OCH_3_), 3.81 (s, 3H, OCH_3_); Anal. Calcd for C_17_H_18_N_2_O_4_: C = 64.96, H = 5.77, N= 8.91, O = 20.36, Found C = 64.94, H = 5.74, N = 8.88, O = 20.35; EI MS *m/z* (% rel. abund.): 314 (M^+^, 90), 137 (40), 135 (100).

*N'-Benzylidene-4-methoxybenzohydrazide* (**20**). Solid, M.p.: 202 °C; ^1^H-NMR (DMSO-*d_6_*): δ 11.74 (s, 1H, NH), 8.44 (s, 1H, N=CH-Ar), 7.92 (d, 2H, *J*_2,6/3,5_ = 8.5 Hz, H-2, H-6), (d, 2H, *J*_3,5/2,6_ = 6.5 Hz, H-5 H-6), 7.48–7.44 (m, 3H, H-3, H-4 ,H-5), 7.08 (d, 2H, *J*_3,5/2,6_ = 8.5 Hz, H-3, H-5), 3.84 (s, 3H, OCH_3_); Anal. Calcd for C_15_H_14_N_2_O_2_: C = 70.85, H = 5.54, N= 11.02, O = 12.58, Found C = 70.86, H = 5.55, N = 11.01, O = 12.57; EI MS *m/z* (% rel. abund.): 254 (M^+^, 70), 135 (100), 77 (30).

*Methyl 4-((4-methoxybenzoylimino)methyl)benzoate* (**21**). Solid, M.p.: 206 °C; ^1^H-NMR (DMSO-*d_6_*): δ 11.92 (s, 1H, NH), 8.50 (s, 1H, N=CH-Ar), 8.04 (d, 2H, *J*_2,6/3,5_ = 8.0 Hz, H-2/H-6), 7.94 (d, 2H, *J*_2,6/3,5_ = 8.5 Hz, H-2, H-6), 7.87 (d, 2H, *J*_3,5/2,6_ = 8.0 Hz, H-3/H-5), 7.09 (d, 2H, *J*_3,5/2,6_ = 8.5 Hz, H-3, H-5), 3.88 (s, 3H, OCH_3_), 3.84 (s, 3H, OCH_3_); Anal. Calcd for C_17_H_16_N_2_O_4_: C = 65.38, H = 5.16, N= 8.97, O = 20.49, Found C = 65.36, H = 5.15, N = 8.94, O = 20.47; EI MS *m/z* (% rel. abund.): 312 (M^+^, 44), 135 (100), 76 (30).

*N'-(4-Fluorobenzylidene)-4-methoxybenzohydrazide* (**22**). Solid, M.p.: 186 °C; ^1^H-NMR (DMSO-*d_6_*): δ 11.76 (s, 1H, NH), 8.44 (s, 1H, N=CH-Ar), 7.92 (d, 2H, *J*_2,6/3,5_ = 8.5 Hz, H-2, H-6), 7.80 (t, 2H, *J*_2,6/2,6,F_ = 7.0 Hz, H-2/H-6), 7.32 (t, 2H, *J*_3,5/2,6,F_ = 7.0 Hz, H-2/H-6), 7.07 (d, 2H, *J*_3,5/2,6_ = 8.5 Hz, H-3, H-5), 3.84 (s, 3H, OCH_3_); Anal. Calcd for C_15_H_13_FN_2_O_2_: C = 66.17, H = 4.81, F = 6.98, N = 10.29, O = 11.75, Found C = 66.13, H = 4.79, F = 6.95, N = 10.27, O = 11.73; EI MS *m/z* (% rel. abund.): 272 (M^+^, 78), 135 (100), 95 (30).

*N'-(3-Methoxybenzylidene)-4-methoxybenzohydrazide* (**23**). Solid, M.p.: 121.6 °C; ^1^H-NMR (DMSO-*d_6_*): δ 11.74 (s, 1H, NH), 8.41 (s, 1H, N=CH-Ar), 7.92 (d, 2H, *J*_2,6/3,5_ = 9.0 Hz, H-2, H-6), 7.40 (t, 1H, *J*_5(4,6)_ = 7.5 Hz, H-5), 7.30–725 (m, 1H, H-4), 7.07 (d, 2H, *J*_3,5/2,6_ = 9.0 Hz, H-3, H-5), 7.81 (dd, 1H, *J*_6,5_ = 7.5 Hz, *J*_6,4 _= 2.0 Hz, H-6), 3.84 (s, 3H, OCH_3_), 3.81 (s, 3H, OCH_3_); Anal. Calcd for C_16_H_16_N_2_O_3_: C = 67.59, H = 5.67, N= 9.85, O = 16.88, Found C = 67.57, H = 5.64, N = 9.82, O = 16.85; EI MS *m/z* (% rel. abund.): 284 (M^+^, 55), 135 (100), 107 (30).

*N'-(4-Methoxybenzylidene)-4-methoxybenzohydrazide* (**24**). Solid, M.p.: 174 °C; ^1^H-NMR (DMSO-*d_6_*): δ 11.62 (s, 1H, NH), 8.37 (s, 1H, N=CH-Ar), 7.91(d, 2H, *J*_2,6/3,5_ = 8.5 Hz, H-2, H-6), 7.68 (d, 2H, *J*_2,6/3,5_ = 8.0 Hz, H-2, H-6), 7.07 (d, 2H, *J*_3,5/2,6_ = 9.0 Hz, H-3, H-5), 7.03(d, 2H, *J*_3,5/2,6_ = 8.0 Hz, H-3, H-5), 3.84 (s, 3H, OCH_3_), 3.81 (s, 3H, OCH_3_); Anal. Calcd for C_16_H_16_N_2_O_3_: C = 67.59, H = 5.67, N= 9.85, O = 16.88, Found C = 67.57, H = 5.64, N = 9.82, O = 16.85; EI MS *m/z* (% rel. abund.): 284 (M^+^, 85), 135 (100), 95 (40).

*N'-(4-Chlorobenzylidene)-4-methoxybenzohydrazide* (**25**). Solid, M.p.: 198 °C; ^1^H-NMR (DMSO-*d_6_*): δ 11.80 (s, 1H, NH), 8.37 (s, 1H, N=CH-Ar), 7.912 (d, 2H, *J*_2,6/3,5_ = 9.0 Hz, H-2, H-6), 7.76 (d, 2H, *J*_2,6/3,5_ = 8.5 Hz, H-2, H-6), 7.54 (d, 2H, *J*_3,5/2,6_ = 8.5 Hz, H-3, H-5), 7.07 (d, 2H, *J*_3,5/ 2,6_ = 9.0 Hz, H-3, H-5), 3.84 (s, 3H, OCH_3_), 3.85 (s, 3H, OCH_3_); Anal. Calcd for C_15_H_13_ClN_2_O_2_: C = 62.40, H = 4.54, N= 9.70, O = 11.08, Found C = 62.41, H = 4.53, N = 9.71, O = 11.06; EI MS *m/z* (% rel. abund.): 290 (M^+^+2, 100), 288 (M^+^, 32), 135 (100), 113 (15), 111 (50).

*4-Methoxy-N'-(thiophen-2-methylene)benzohydrazide* (**26**). Solid, M.p.: 209 °C; ^1^H-NMR (DMSO-*d_6_*): δ 11.67 (s, 1H, NH), 8.65 (s, 1H, N=CH-Ar), 7.90 (d, 2H, *J*_2,6/3,5_ = 9.0 Hz, H-2, H-6), 7.65 (d, 1H, *J*_3,4_ = 5.0 Hz, H-3), 7.45 (d, 1H, *J*_5,4 _= 3.0 Hz, H-5), 7.15 (d, 1H, *J*_4,5 _= 5.0, *J*_4,3 _= 3.0 Hz, H-4), 7.06 (d, 2H, *J*_3,5/2,6_ = 9.0 Hz, H-3, H-5), 3.84 (s, 3H, OCH_3_); Anal. Calcd for C_13_H_12_N_2_O_2_S: C = 59.98, H = 4.65, N= 10.76, O = 12.29, S = 12.32, Found C = 59.96, H = 4.63, N = 10.74, O = 12.27, S = 12.30; EI MS *m/z* (% rel. abund.): 260 (M^+^, 65), 135 (100), 83 (28).

*N'-(3-Bromo-4-hydroxybenzylidiene)-2-methoxybenzohydrazide* (**27**). Solid, M.p.: 209 °C; ^1^H-NMR (DMSO-*d_6_*): δ 11.67 (s, 1H, NH), 10.83 (s, 1H, OH), 8.30 (s, 1H, N=CH-Ar), 7.91 (d, 2H, *J*_2,6/3,5_ = 8.5 Hz, H-2, H-6), 7.86 (s, 1H, H-2), 7.56 (d, 1H, *J*_6,5_ = 8.0 Hz, H-6), 7.06 (d, 2H, *J*_3,5/2,6_ = 9.0 Hz, H-3, H-5), 7.03 (d, 1H, *J*_5,6_ = 8.0 Hz, H-5), 3.84 (s, 3H, OCH_3_); Anal. Calcd for C_15_H_13_BrN_2_O_3_: C = 51.60, H = 3.75, Br = 22.88, N= 8.02, O = 13.75, Found C = 51.57, H = 3.73, Br = 22.85, N = 7.99, O = 13.73; EI MS *m/z* (% rel. abund.): 350 (M+2, 56), 348 (M^+^, 57), 172 (26), 170 (25), 135 (100), 92 (20).

*N'-(3-Hydroxy-2-iodo-4-methoxybenzylidene)-4-methoxybenzohydrazide* (**28**). Solid, M.p. = 147 °C; ^1^H-NMR (DMSO-*d_6_*): δ 11.65 (s, 1H, NH), 9.72 (s, 1H, OH), 8.68 (s, 1H, N=CH-Ar), 7.93 (d, 2H, *J*_2,6/3,5_ = 8.5 Hz, H-2, H-6), 7.50 (d, 1H, *J*_6,5_ = 8.0 Hz, H-6), 7.09 (d, 1H, *J*_5,6_ = 8.0 Hz, H-5), 7.06 (d, 2H, *J*_3,5/2,6_ = 8.5 Hz, H-3, H-5), 3.87 (s, 3H, OCH_3_); Anal. Calcd for C_16_H_15_IN_2_O_4_: C = 45.09, H = 3.55, I = 29.78, N= 6.57, O = 15.02, Found C = 45.07, H = 3.53, I = 29.77, N = 6.55, O = 14.99; EI MS *m/z* (% rel. abund.): 426 (M^+^, 15), 299 (36), 248 (20), 135 (100).

*N'-(3,5-Dimethoxybenzylidene)-2-methoxybenzohydrazide* (**29**). Solid, M.p. = 184 °C; ^1^H-NMR (DMSO-*d_6_*): δ 11.78 (s, 1H, NH), 8.36 (s, 1H, N=CH-Ar), 9.92 (d, 2H, *J*_2,6/3,5_ = 9.0 Hz, H-2, H-6), 7.08 (d, 2H, *J*_3,5/2,6_ = 9.0 Hz, H-3, H-5), 6.89 (s, 2H, H-2, H-6), 6.57 (s, 1H, H-4), 3.84 (s, 3H,OCH_3_), 3.79 (s, 6H, OCH_3_); Anal. Calcd for C_17_H_18_N_2_O_4_: C = 64.96, H = 5.77, N = 8.91, O = 20.36, Found C = 64.95, H = 5.77, N = 8.88, O = 20.33; EI MS *m/z* (% rel. abund.): 314 (M^+^, 81), 137 (36), 135 (100).

*N'-(4-Nitrobenzylidiene)-4-methoxybenzohydrazide* (**30**). Solid, M.p. = 240 °C; ^1^H-NMR (DMSO-*d_6_*): δ 12.08 (s, 1H, NH), 8.53 (s, 1H, N=CH-Ar), 8.31 (d, 2H, *J*_2,6/3,5_ = 8.0 Hz, H-2, H-6), 8.00 (d, 2H, *J*_3,5/2,6_ = 8.0 Hz, H-3, H-5), 7.94 (d, 2H, *J*_2,6/3,5_ = 8.5 Hz, H-2, H-6), 7.09 (d, 2H, *J*_3,5/ 2,6_ = 8.5 Hz, H-3, H-5), 3.85 (s, 3H, OCH_3_); Anal. Calcd for C_15_H_13_N_3_O_4_: C = 60.20, H = 4.38, N= 14.04, O = 21.38, Found C = 60.17, H = 4.35, N = 14.02, O = 21.37; EI MS *m/z* (% rel. abund.): 301 (M^+^, 94), 135 (100). 122 (35), 76 (20).

#### 3.2.3. Protocol for Antiglycation Activity

Bovine Serum Albumin (BSA) was purchased from Merck Marker Pvt. Ltd. (Darmstadt, Germany), rutin and methylglyoxal (MG) (40% aqueous solution) were from Sigma Aldrich (Tokyo, Japan), sodium dihydrogen phosphate (NaH_2_PO_4_), disodium hydrogen phosphate (Na_2_HPO_4_) and sodium azide (NaN_3_) were purchased from Scharlau Chemie, S. A. (Barcelona, Spain), while dimethyl sulphoxide (DMSO) was purchased from Fischer Scientific (Loughborough, UK). Bovine Serum Albumin (10 mg/mL), methyl glyoxal (14 mM), various concentrations of the compounds (prepared in DMSO, 10% final concentration), and 0.1 M phosphate buffer (pH 7.4) containing sodium azide (30 mM) was incubated under aseptic conditions at 37 °C for 9 days. After 9 days, each sample was examined for the development of specific fluorescence (excitation, 330 nm; emission, 440 nm) against sample blank [39,57]. Rutin was used as a positive control. All of the experiments were done in a 96-well microplate reader (SpectraMax M2, Molecular Devices, Sunnyvale, CA, USA). The percent inhibition of AGE formation in the test sample versus control was calculated for each inhibitor compound by using the following formula:

% inhibition= (1 − fluorescence of test sample/Fluorescence of the control group) × 100(1)

### 3.3. Software/Statistical

The obtained results were analysed by SoftMaxPro 4.8 and MS-Excel. Results are presented as means ± SEM from three experiments. IC_50_ Values were determined by using EZ-FIT, Enzyme kinetics software by Perrella Scientific, Inc., Hillsborough, NH, USA.

## 4. Conclusions

In conclusion, compounds having hydroxy groups showed good antiglycation activity due to their capacity to inhibit glycoxidation. However, structural modifications can be optimized to achieve the desired activity in this class of compounds.

## Supplementary Materials

Supplementary materials can be accessed at: http://www.mdpi.com/1420-3049/19/1/1302/s1.
