# Supplementary material for: Synthesis of 4-Methoxybenzoylhydrazones and Evaluation of Their Antiglycation Activity
Source: Molecules. 2014 Jan 21;19(1):1286–301. doi: 10.3390/molecules19011286 (PMC6271482; doi:10.3390/molecules19011286)

Supplementary Materials

Figure S1. Compound **30**.

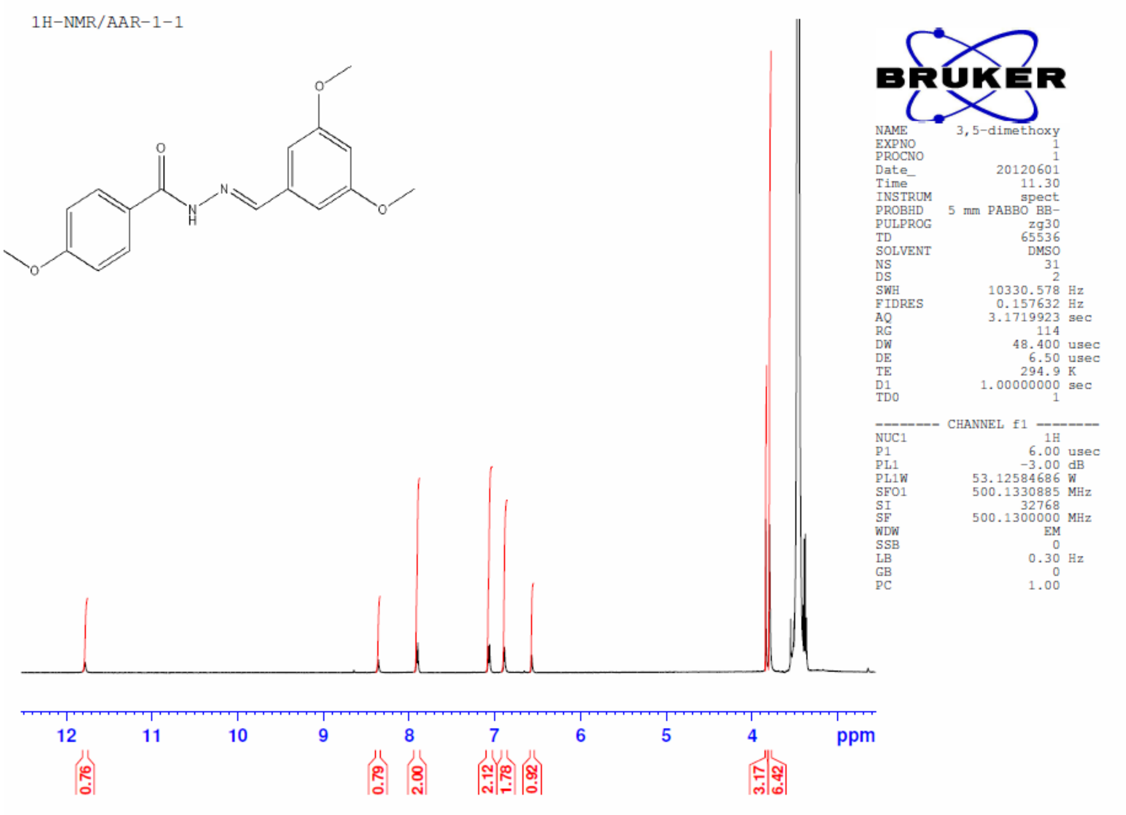

Figure S2. Compound **3**.

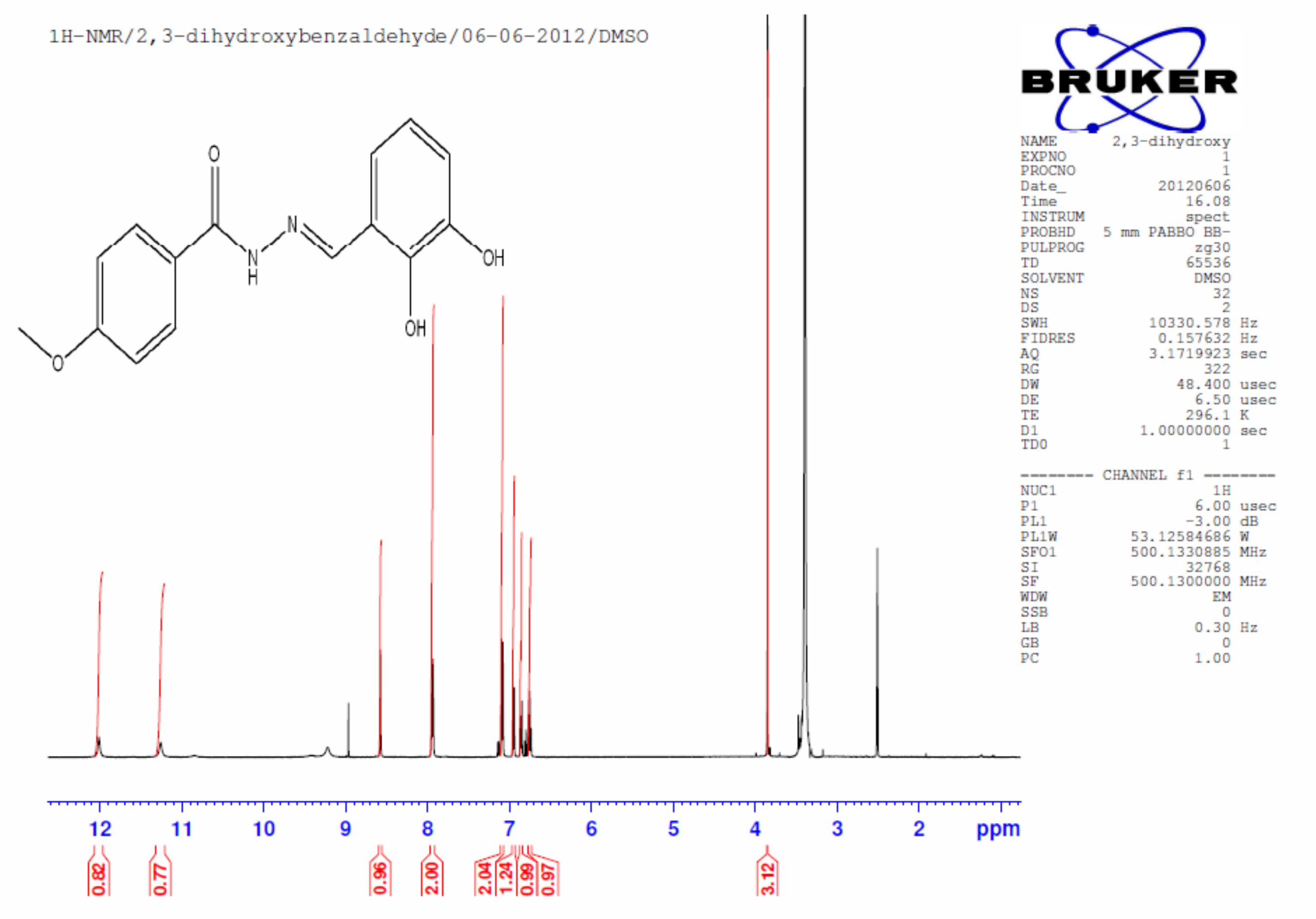

Figure S3. Compound **4**.

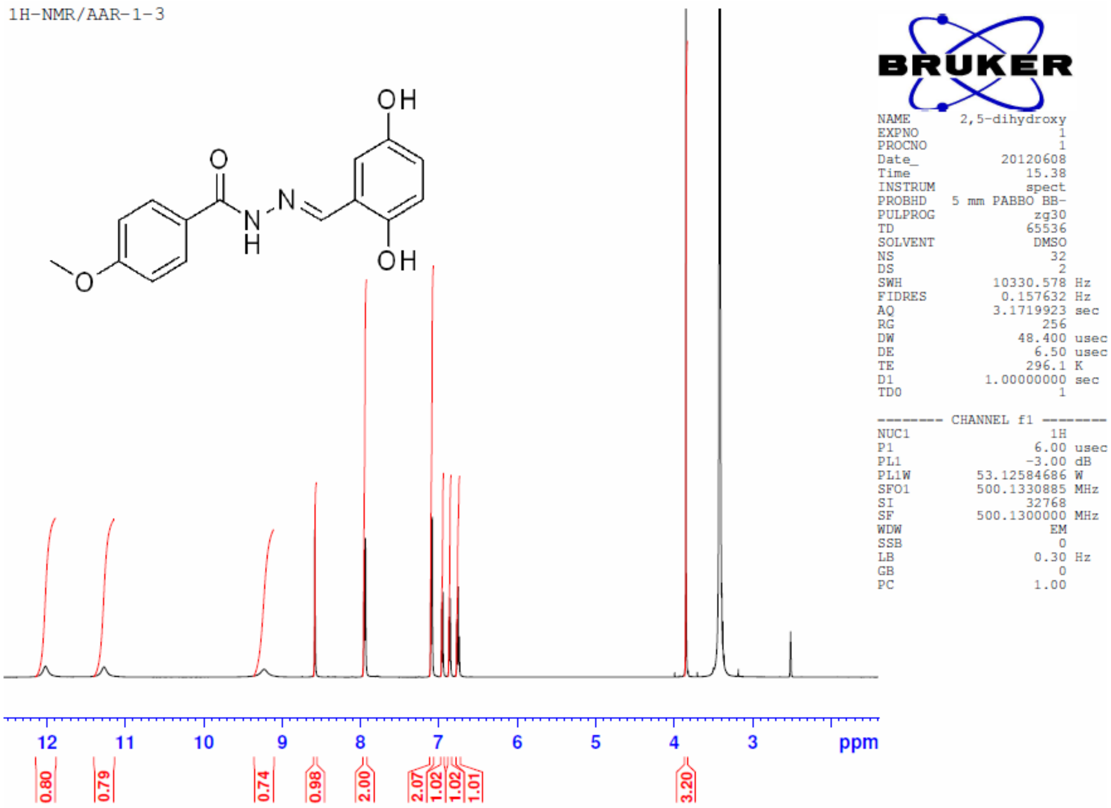

Figure S4. Compound **11**.

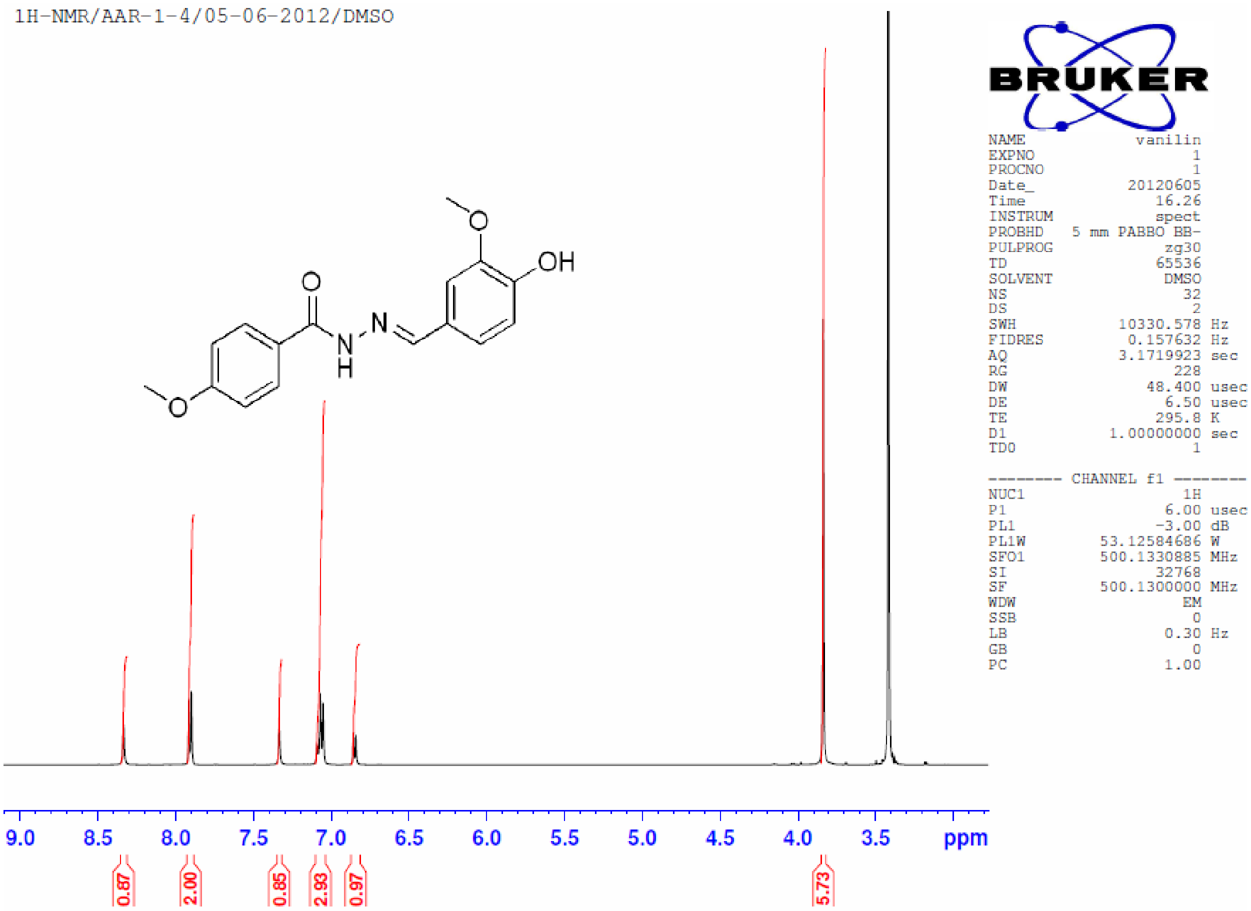

Figure S5. Compound **12**.

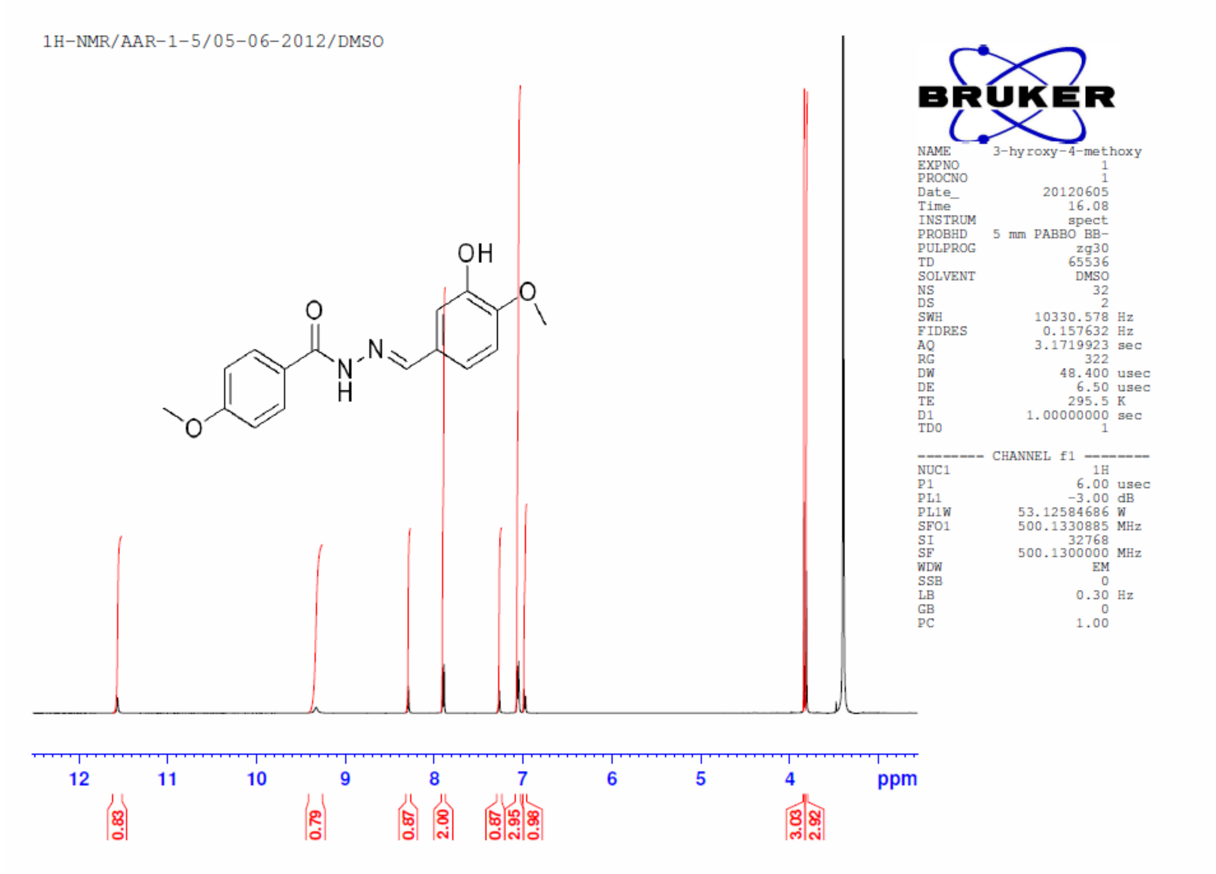

Figure S6. Compound **5**.

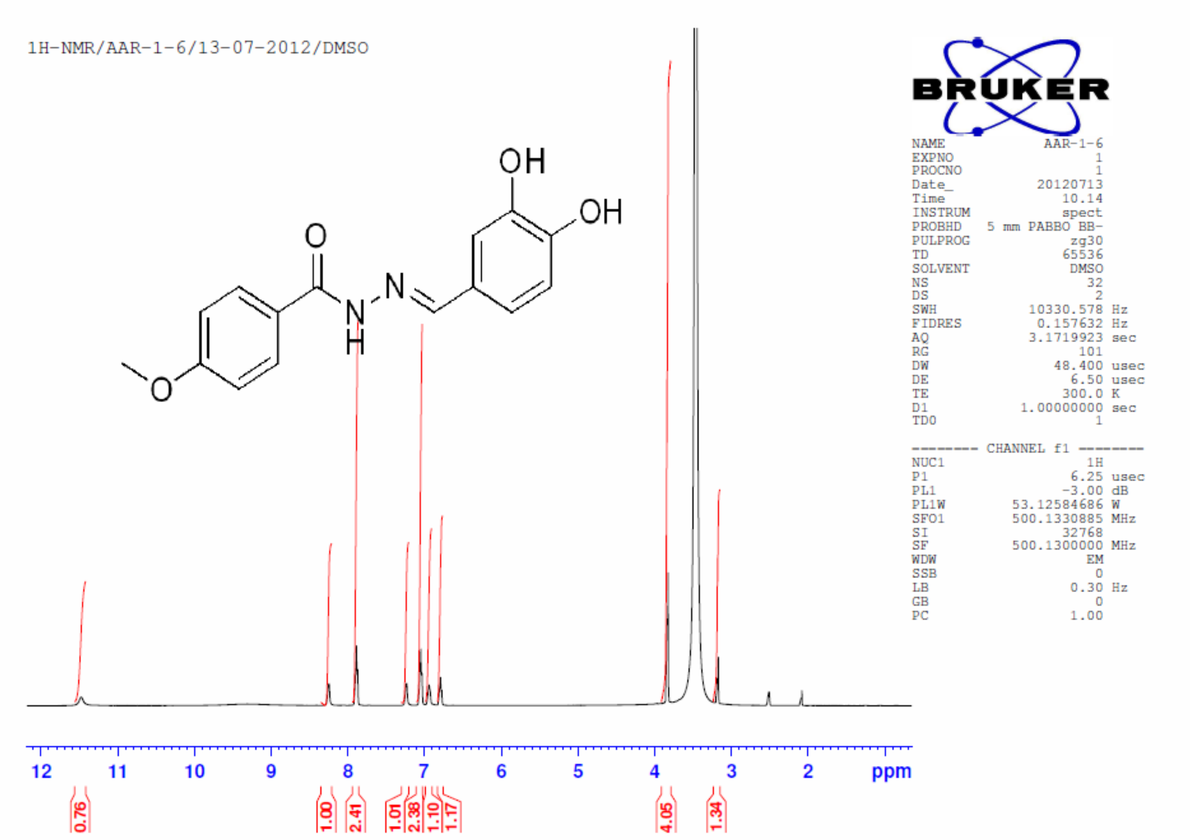

Figure S7. Compound **29**.

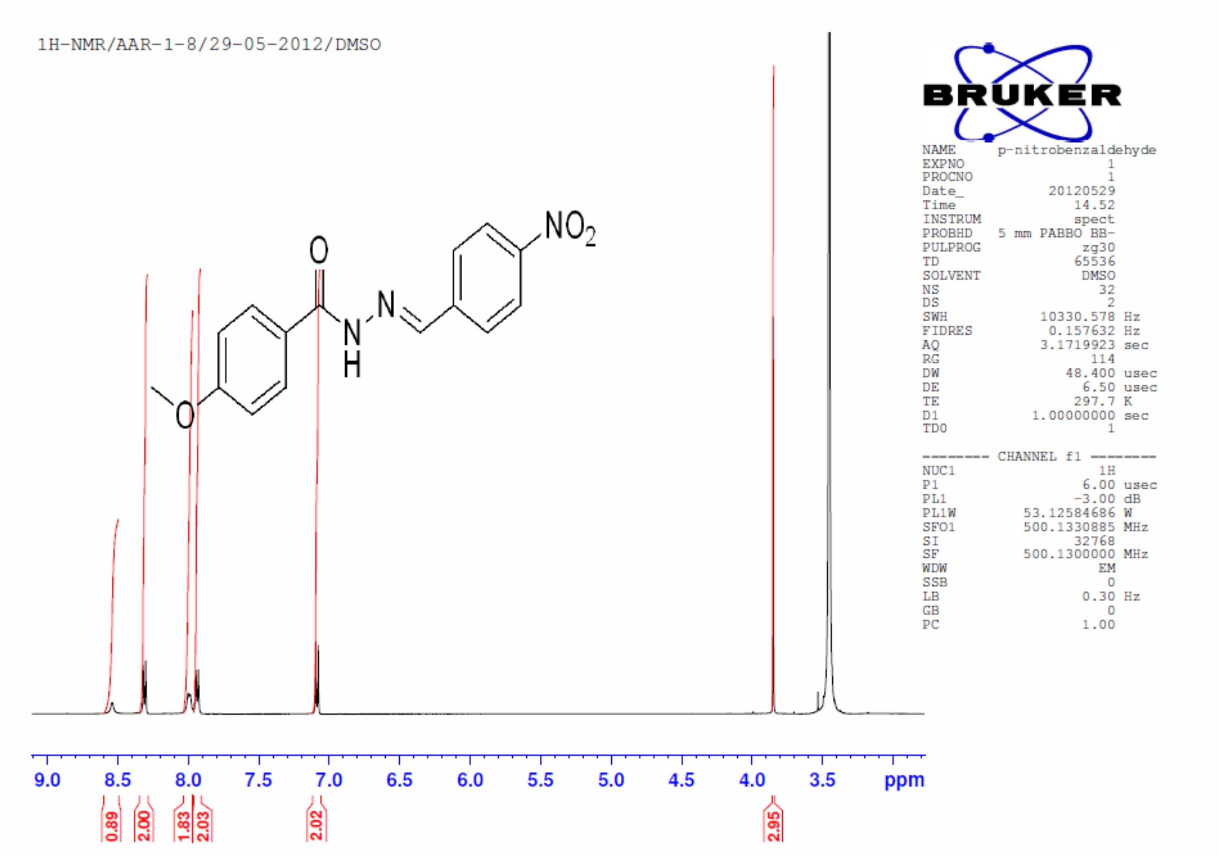

Figure S8. Compound **8**.

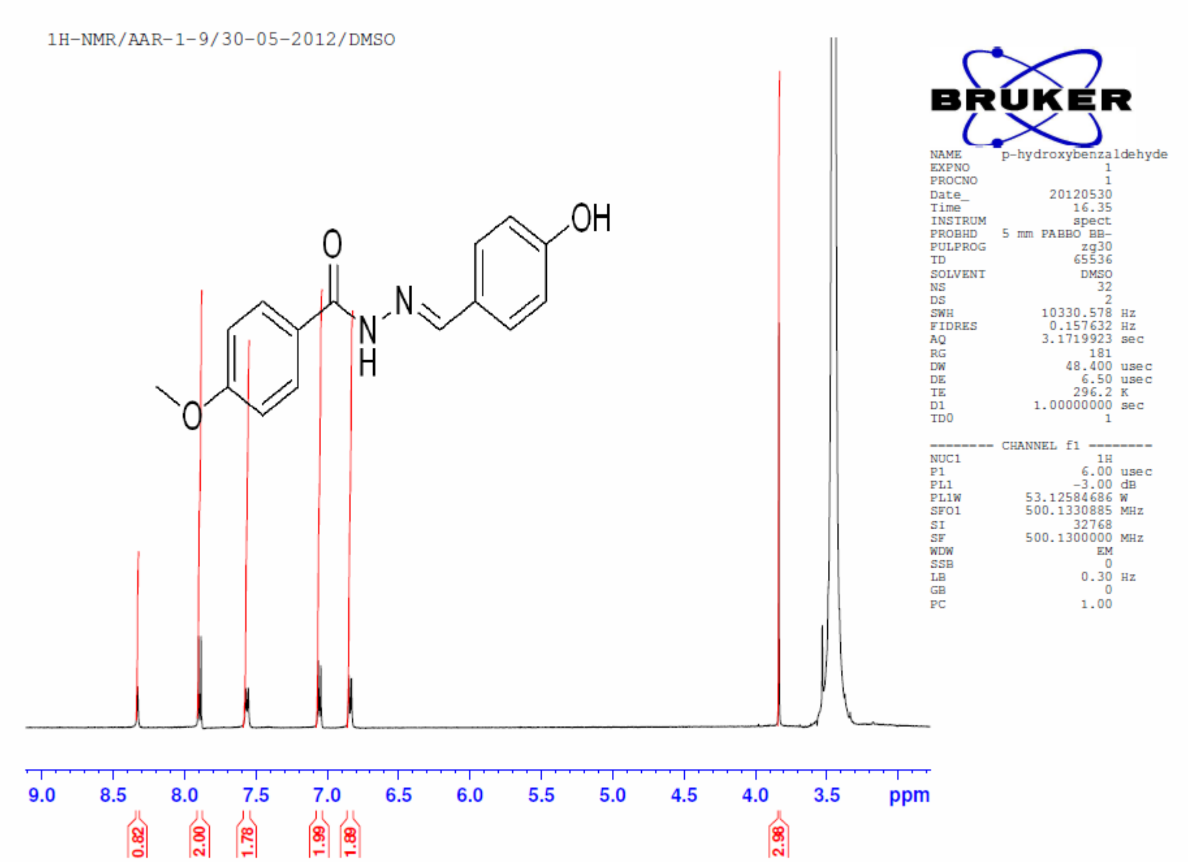

Figure S9. Compound **6**.

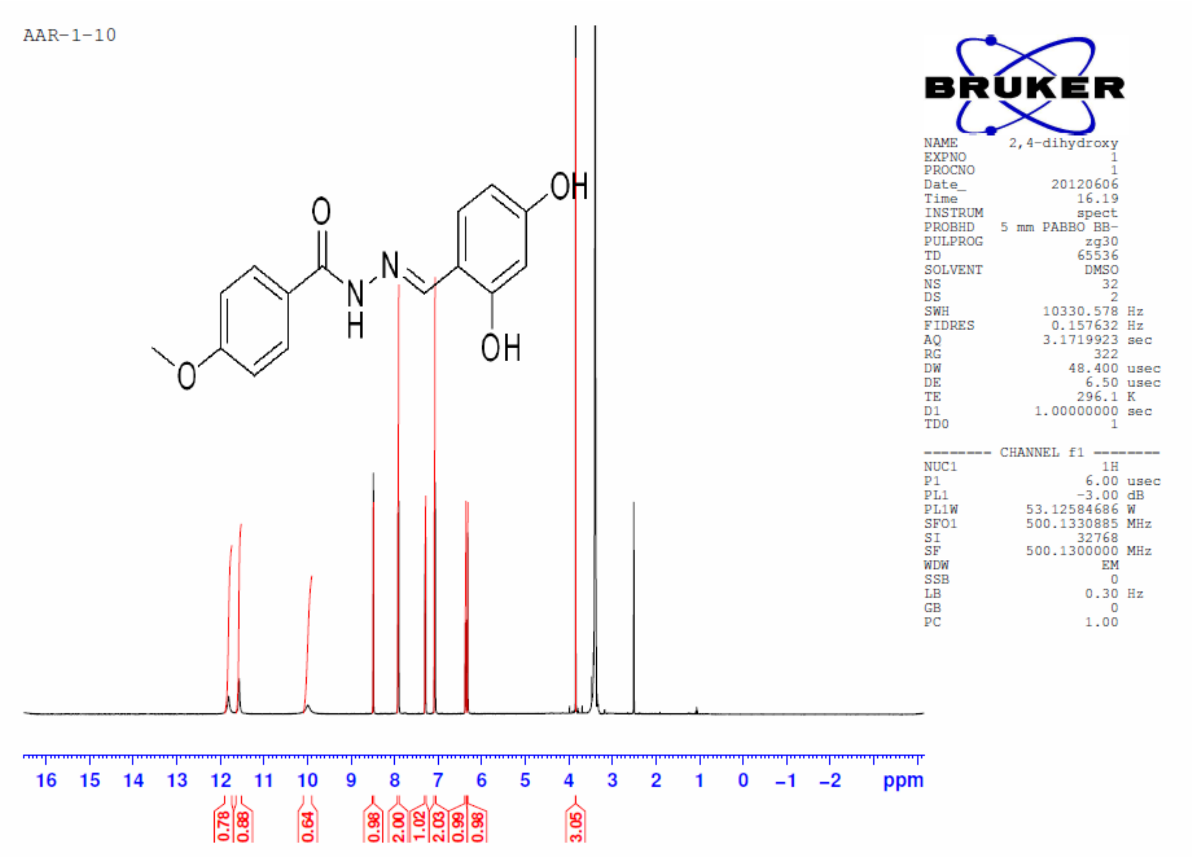

Figure S10. Compound **9**.

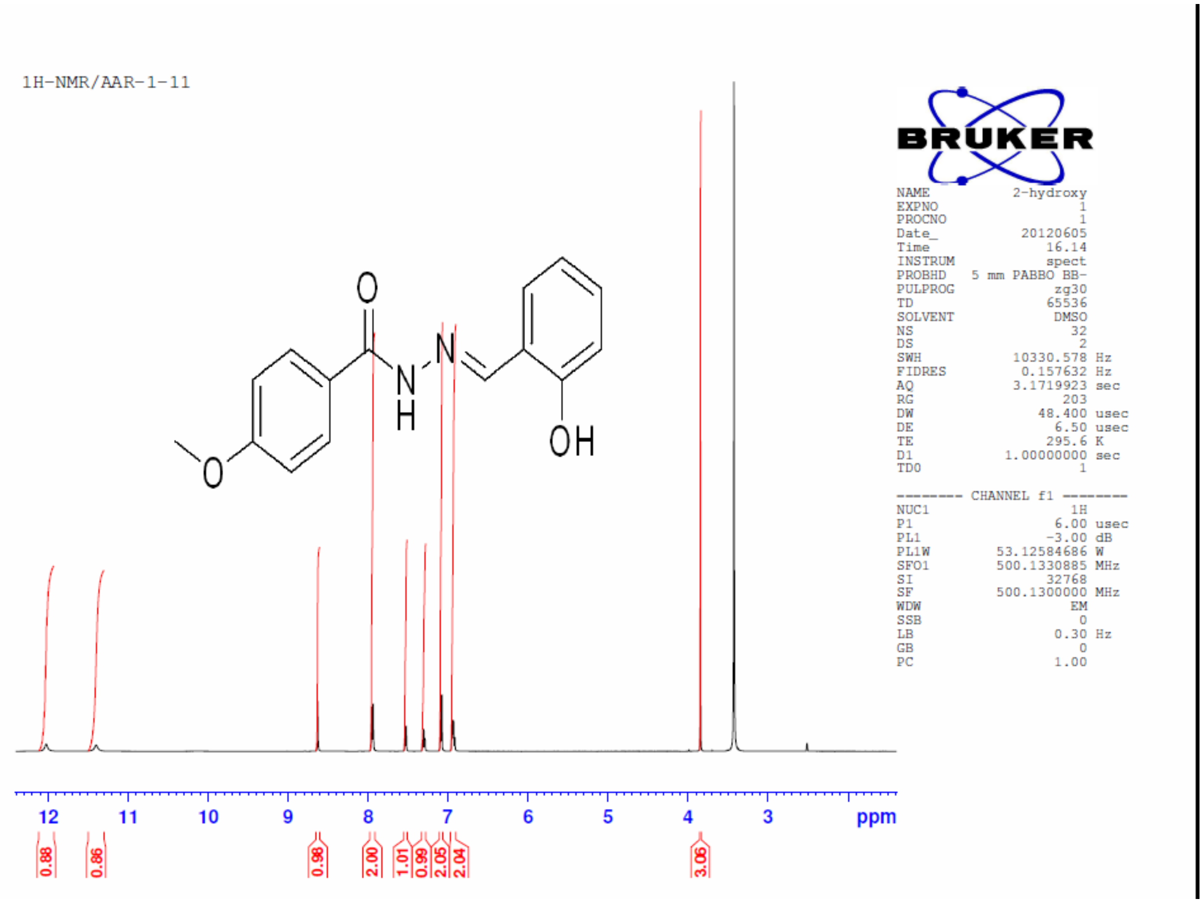

Figure S11. Compound **17**.

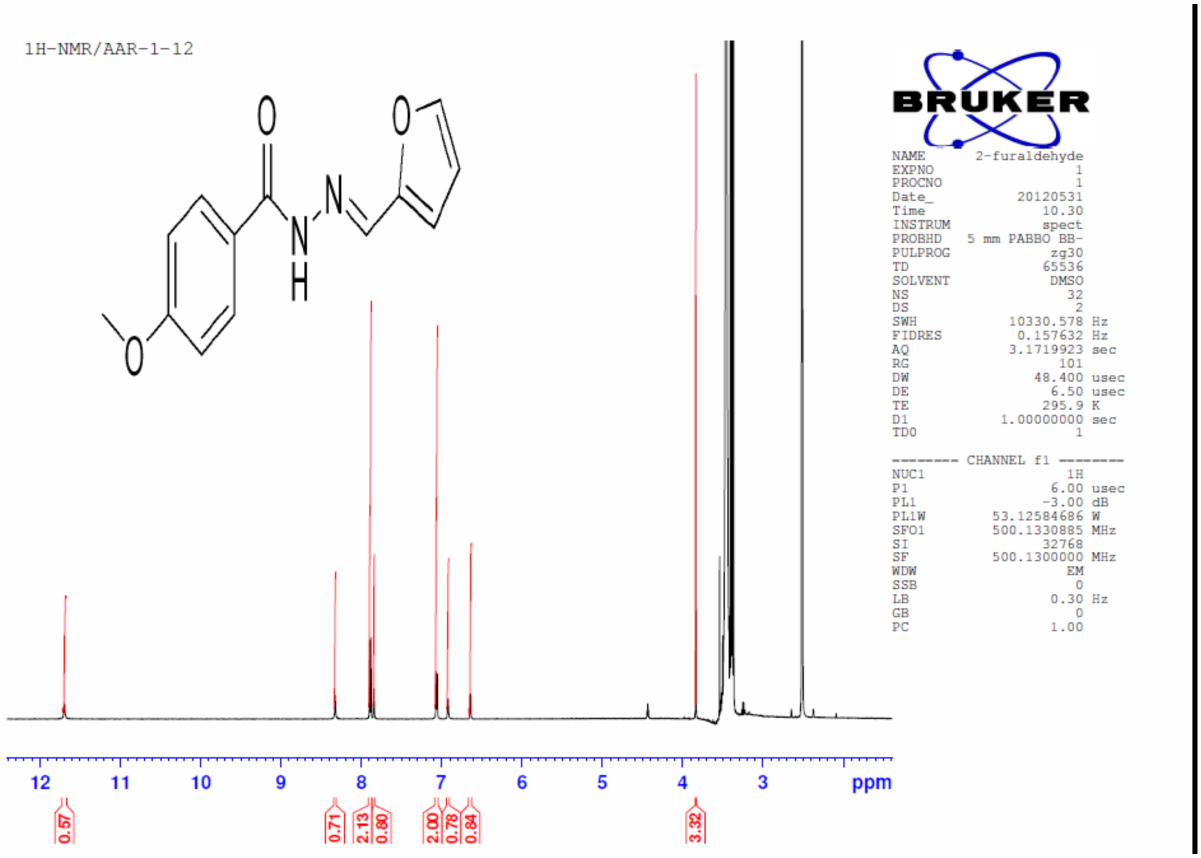

Figure S12. Compound **14**.

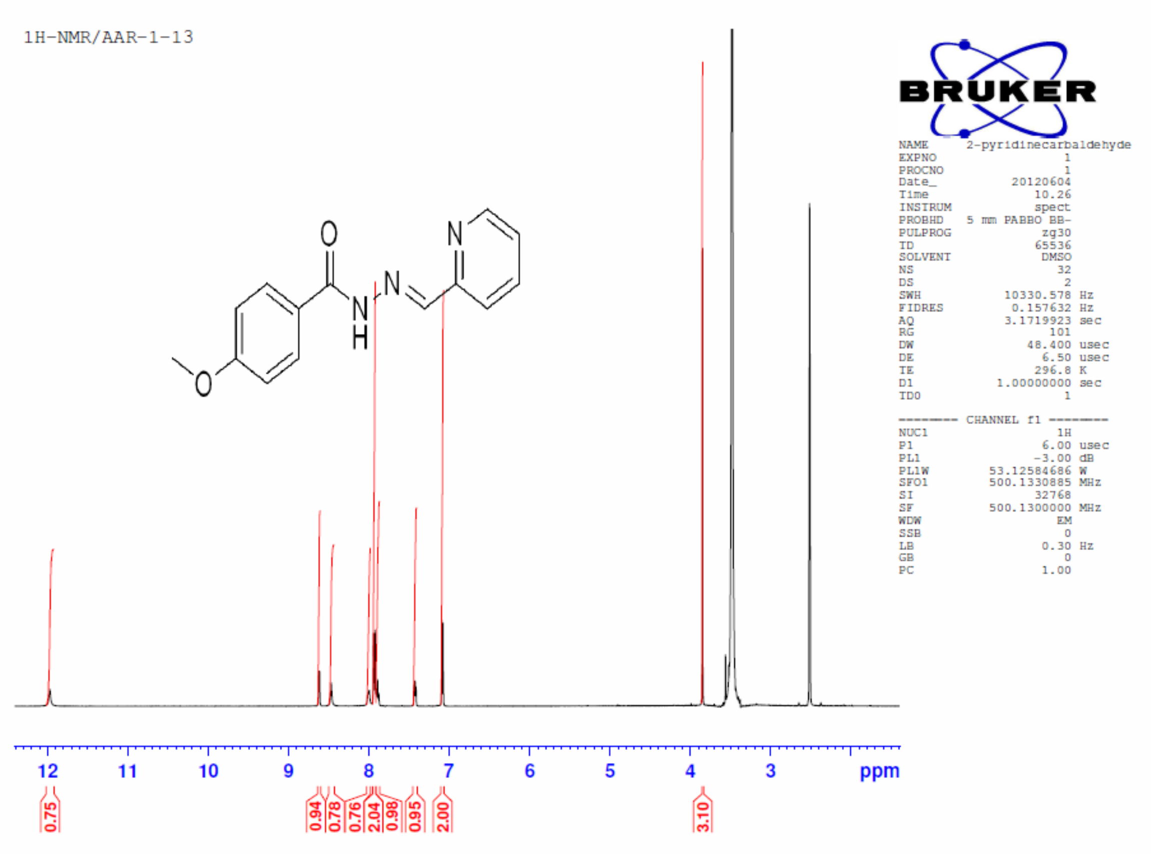

Figure S13. Compound **15**.

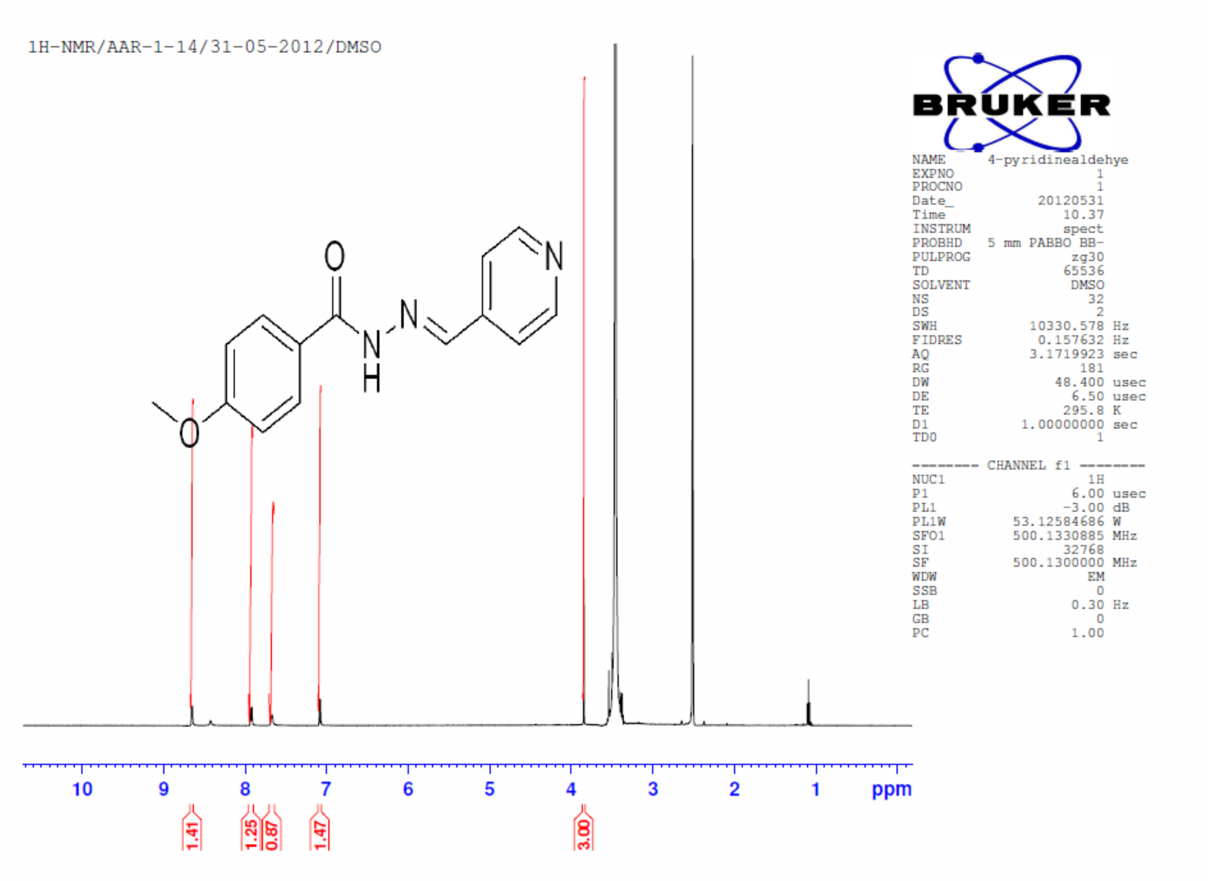

Figure S14. Compound **26**.

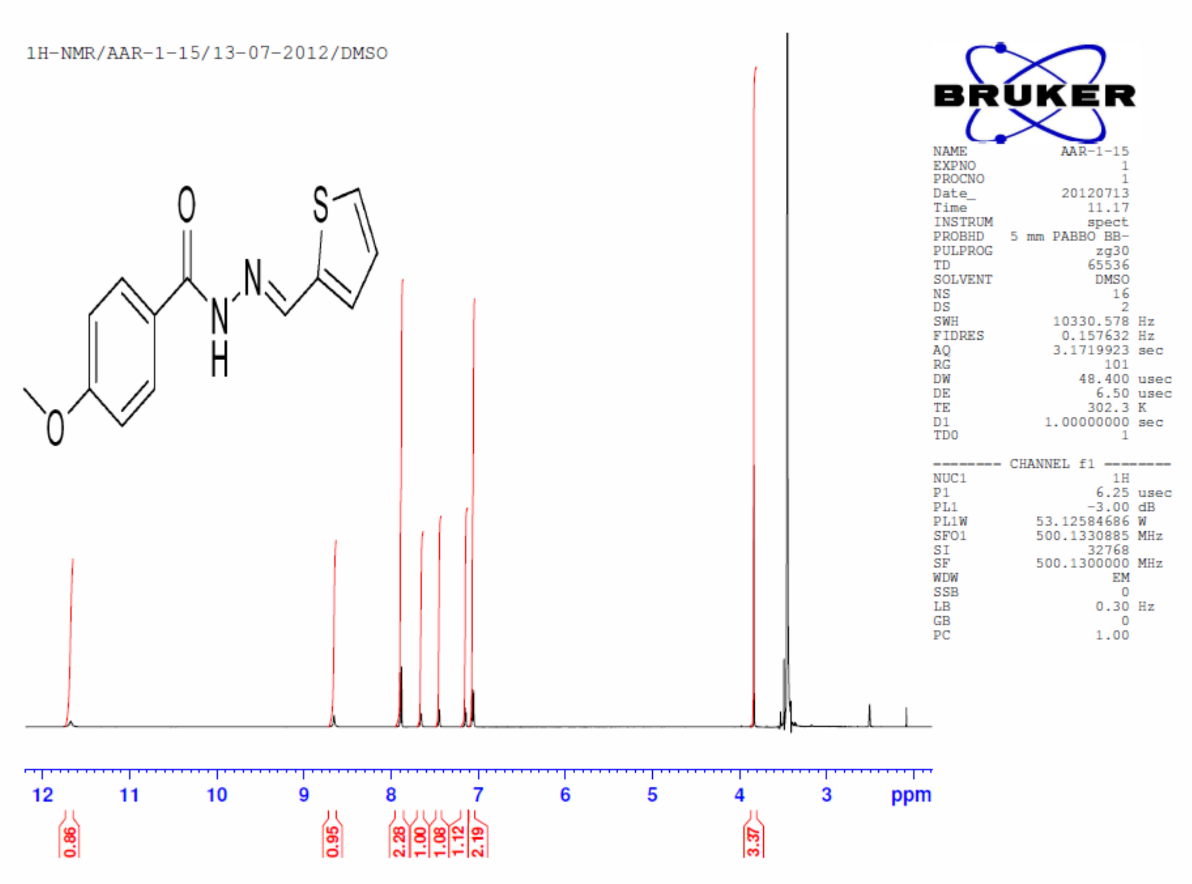

Figure S15. Compound **16**.

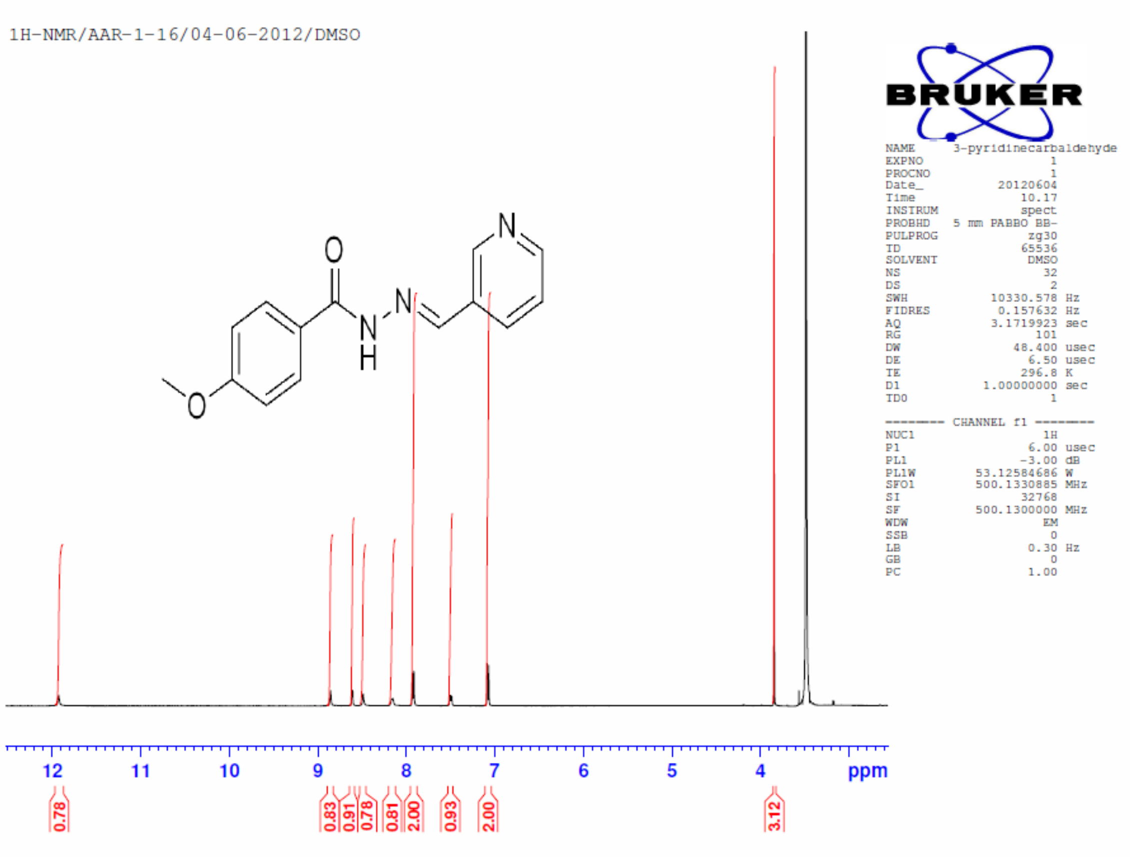

Figure S16. Compound **20**.

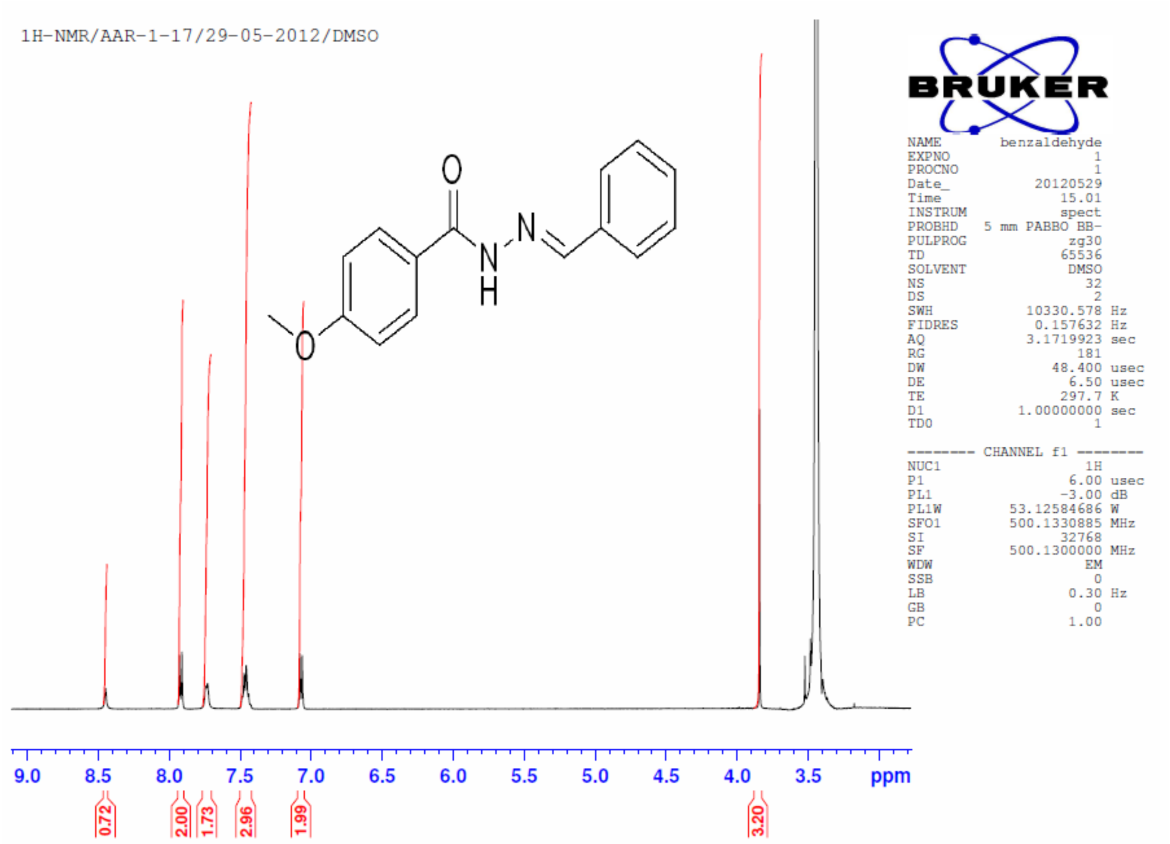

Figure S17. Compound **19**.

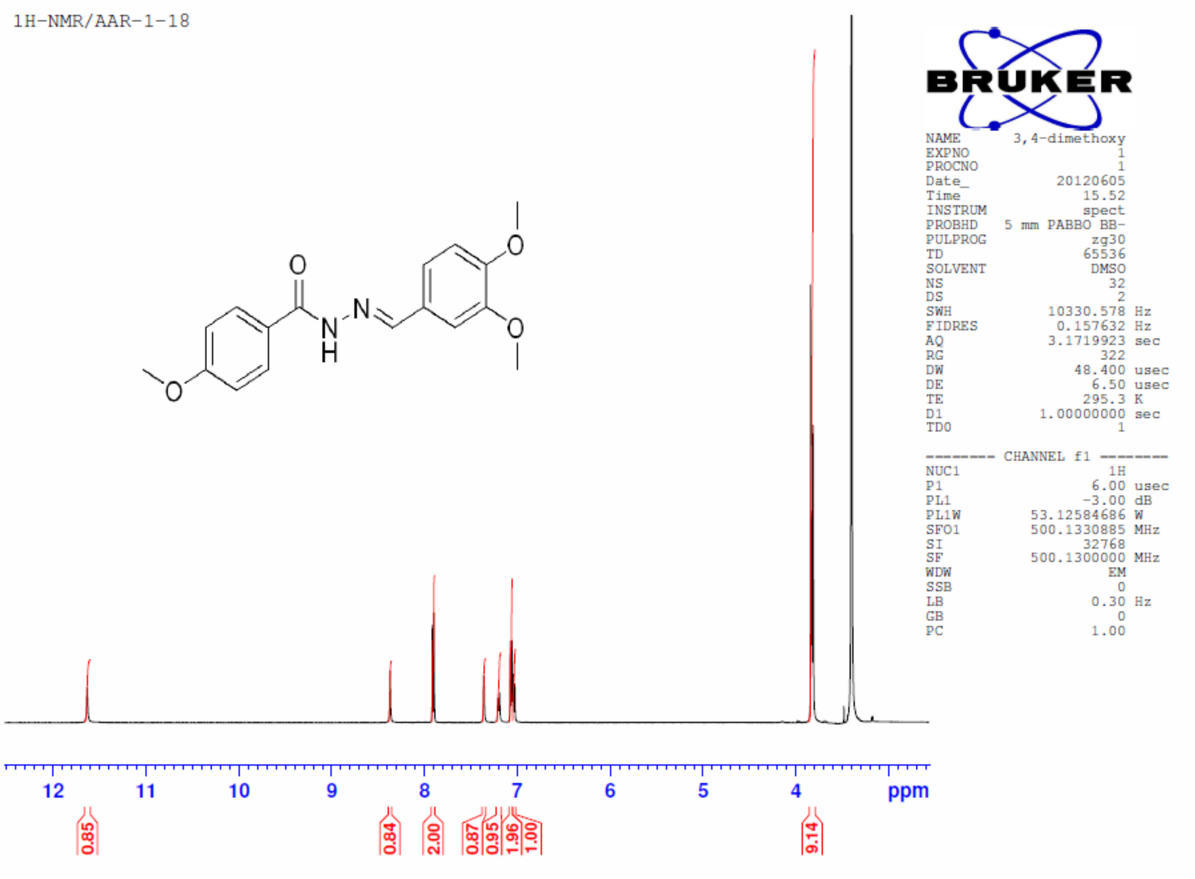

Figure S18. Compound **10**.

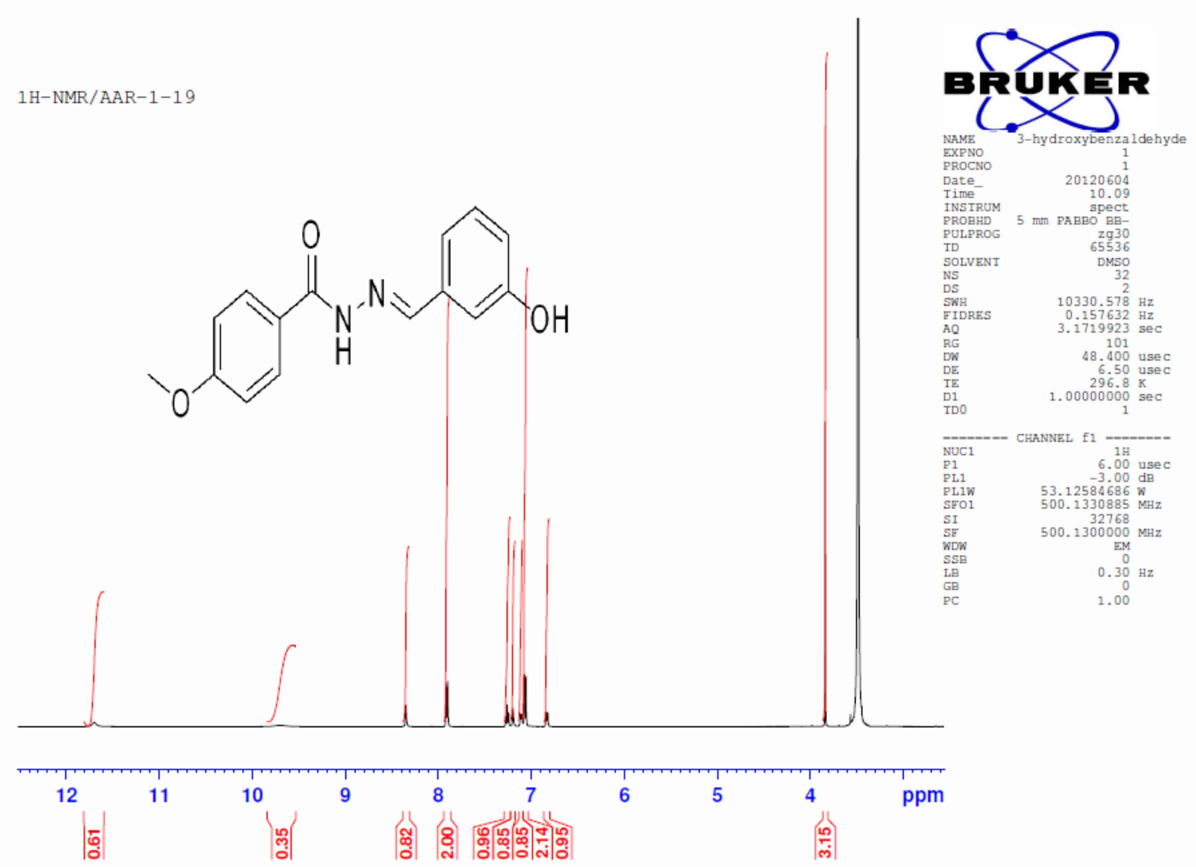

Figure S19. Compound **21**.

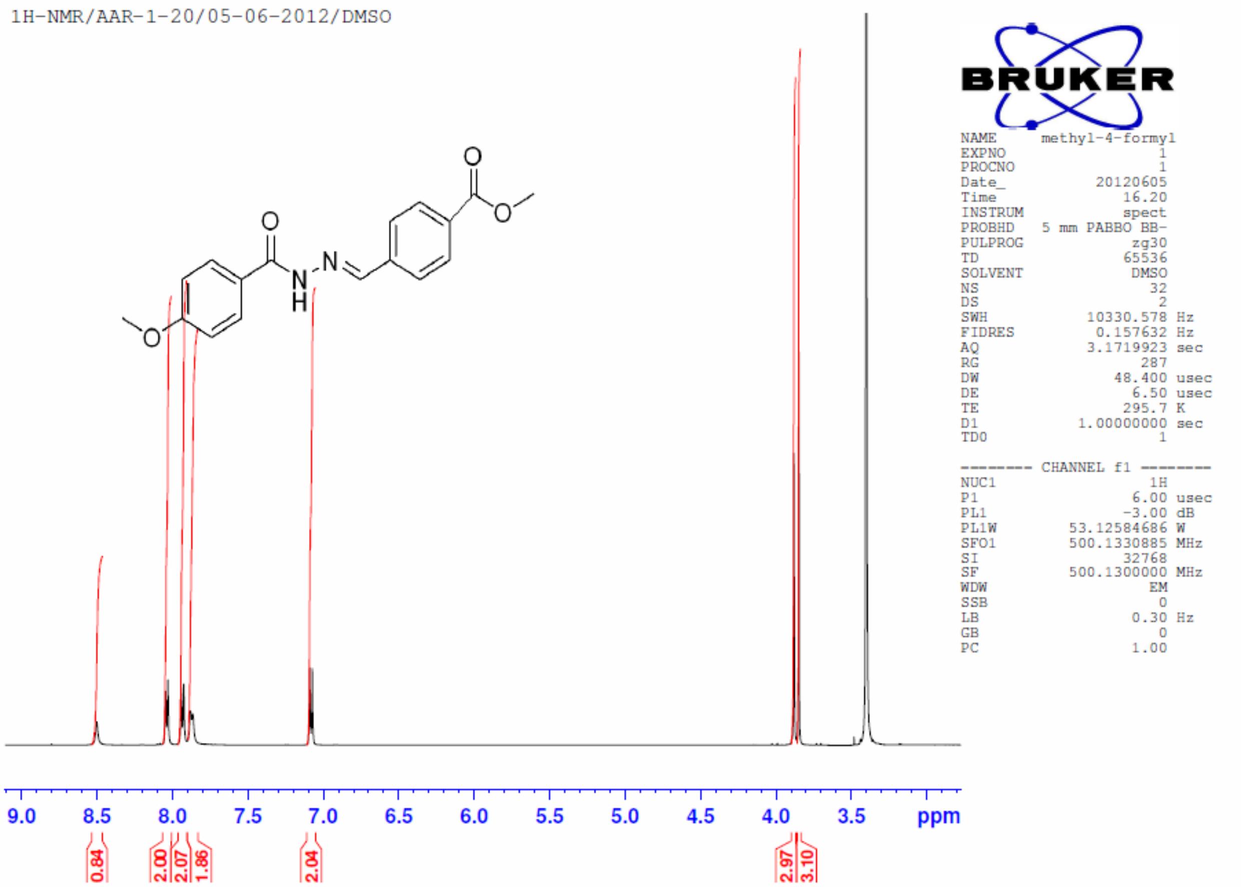

Figure S20. Compound **22**.

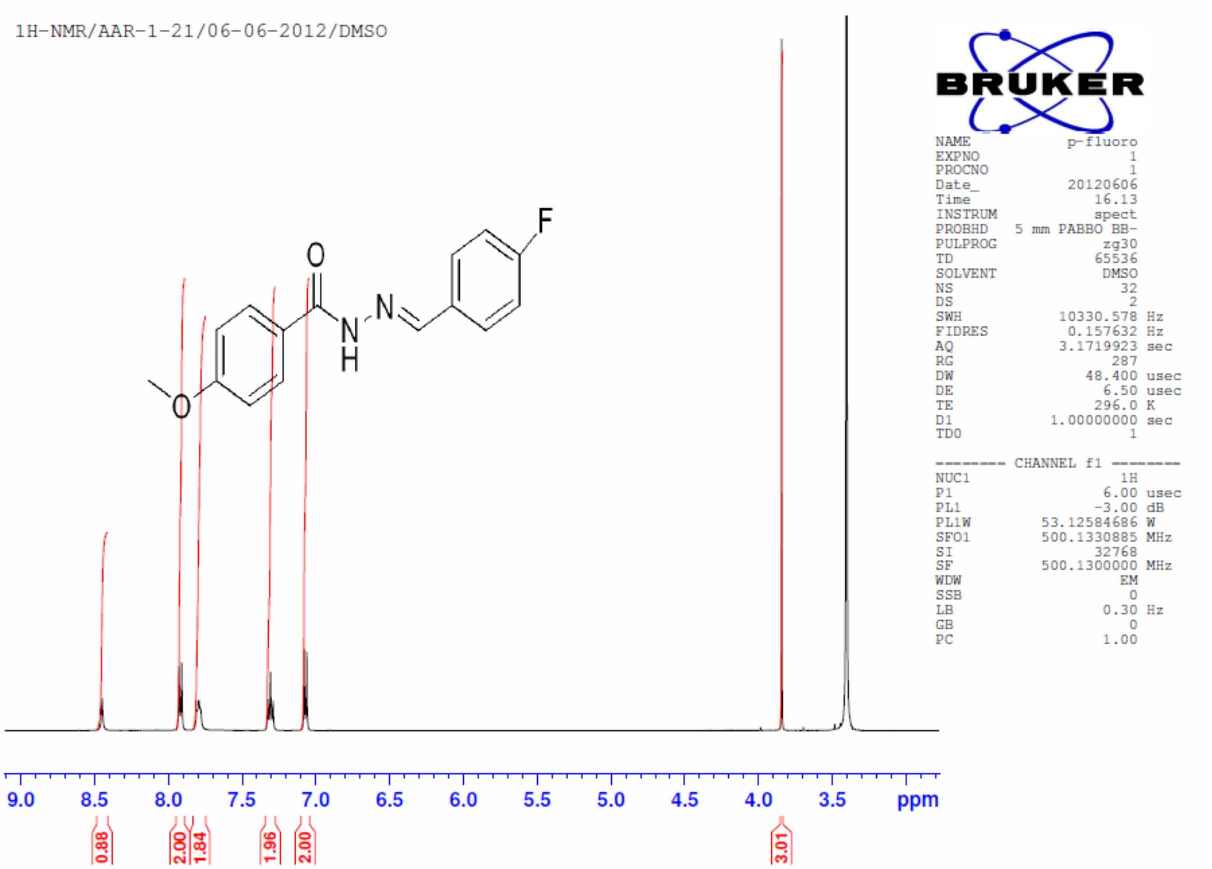

Figure S21. Compound **13**.

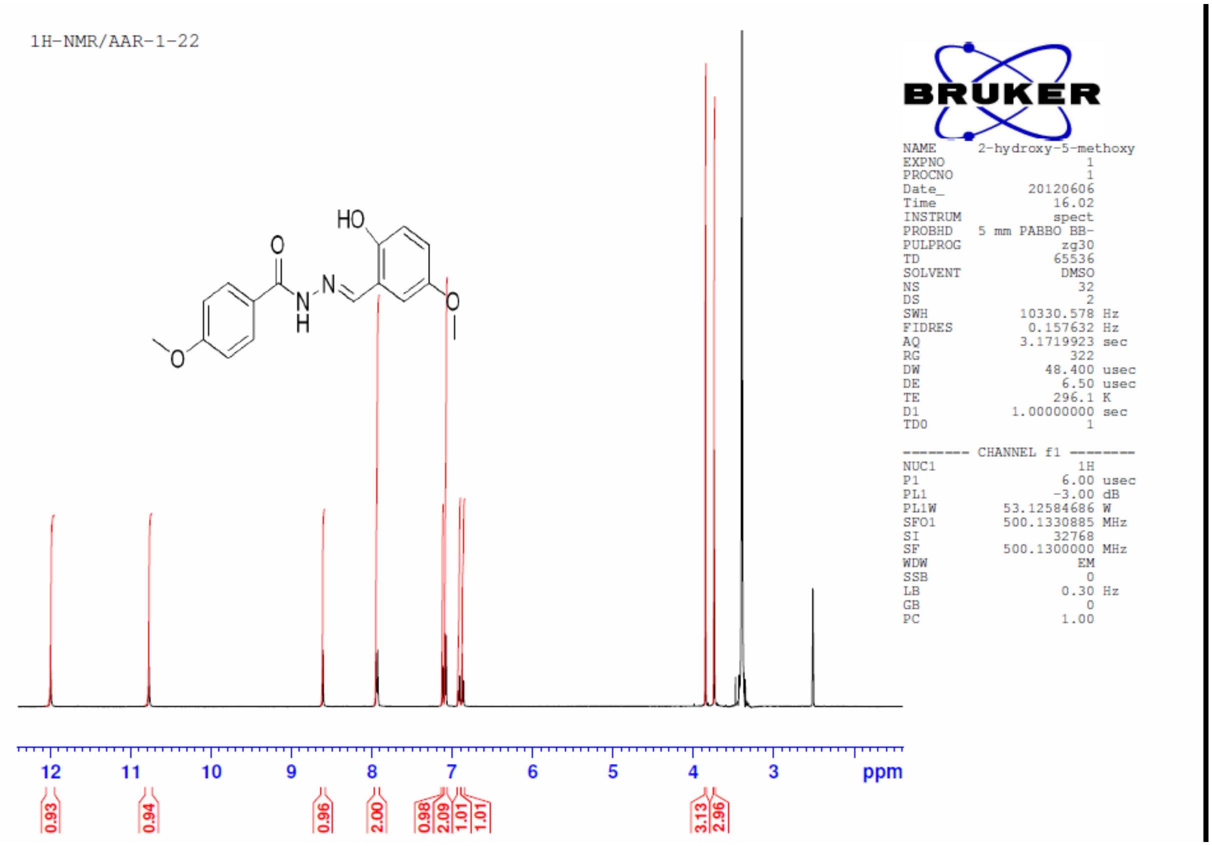

Figure S22. Compound **23**.

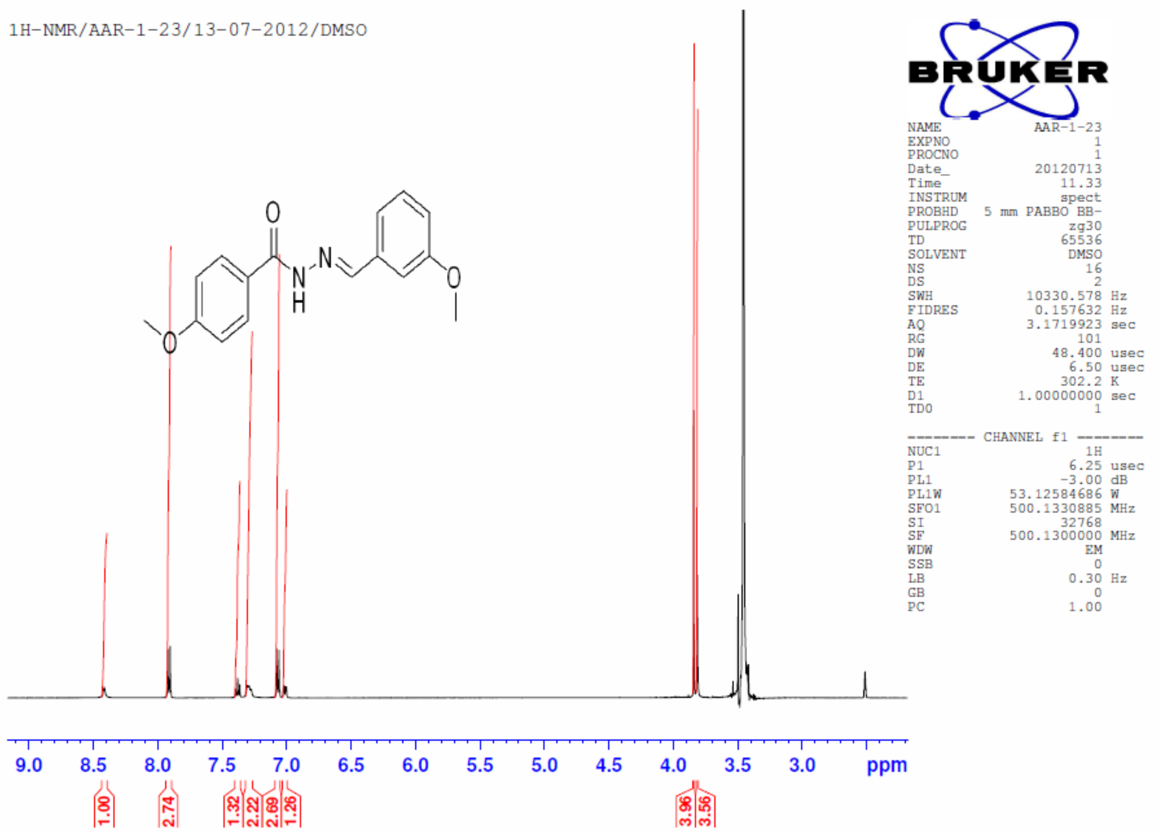

Figure S23. Compound **24**.

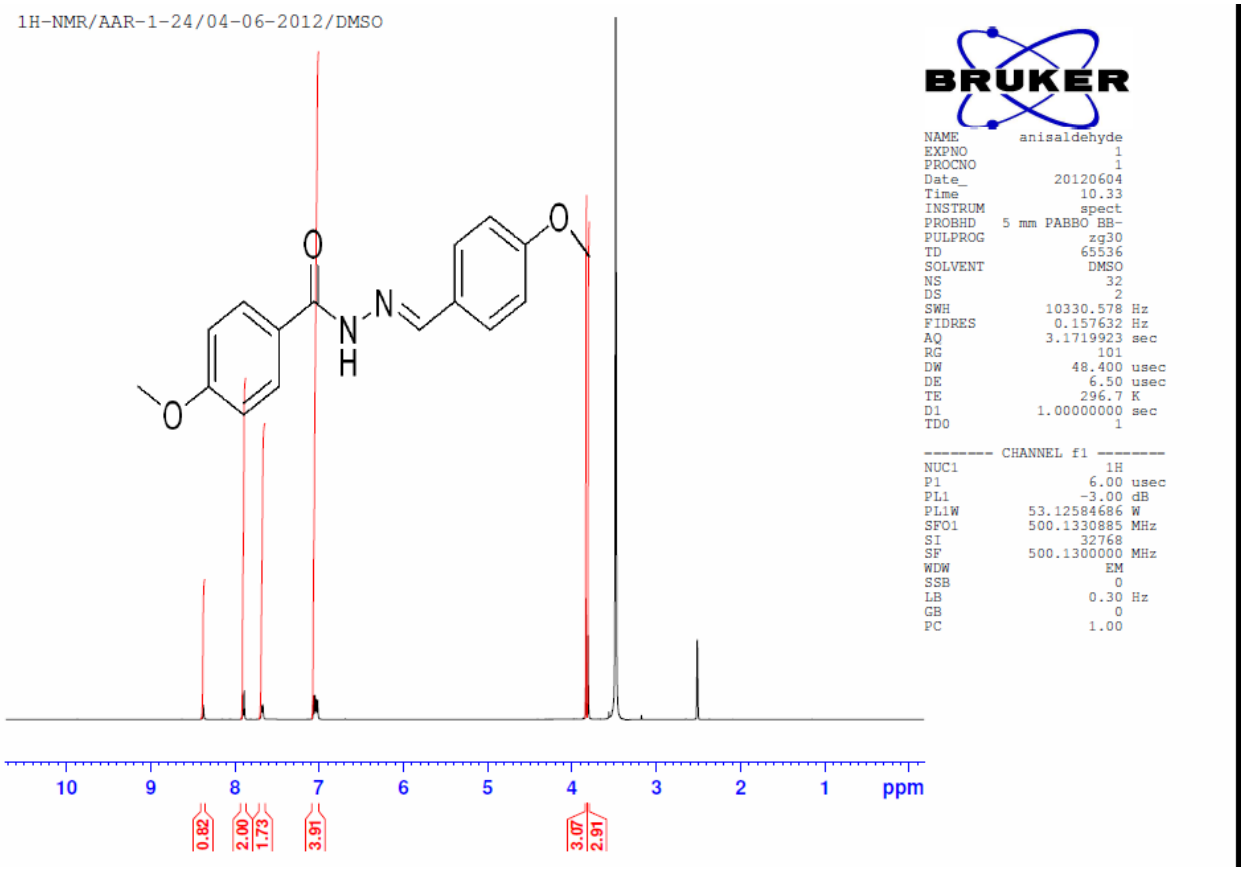

Figure S24. Compound **25**.

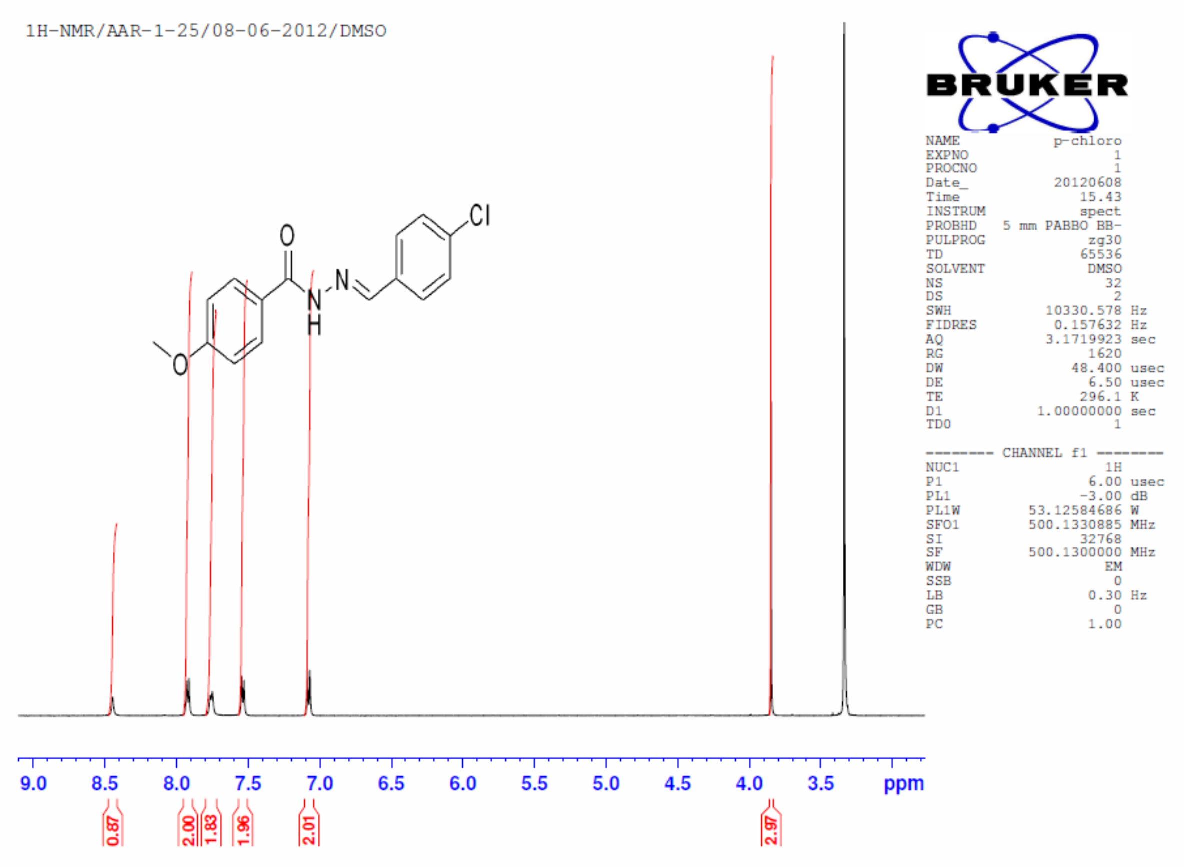

Figure S25. Compound **7**.

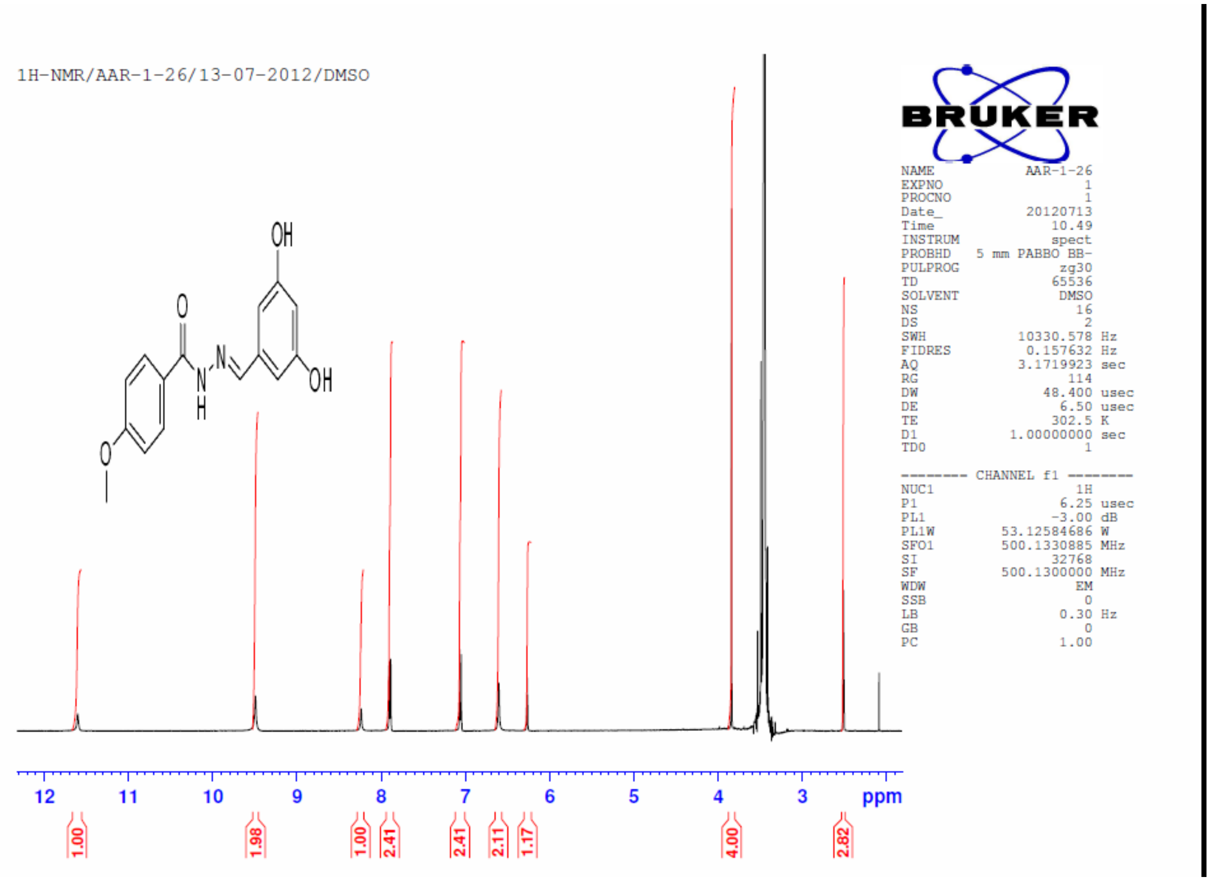

Figure S26. Compound **18**.

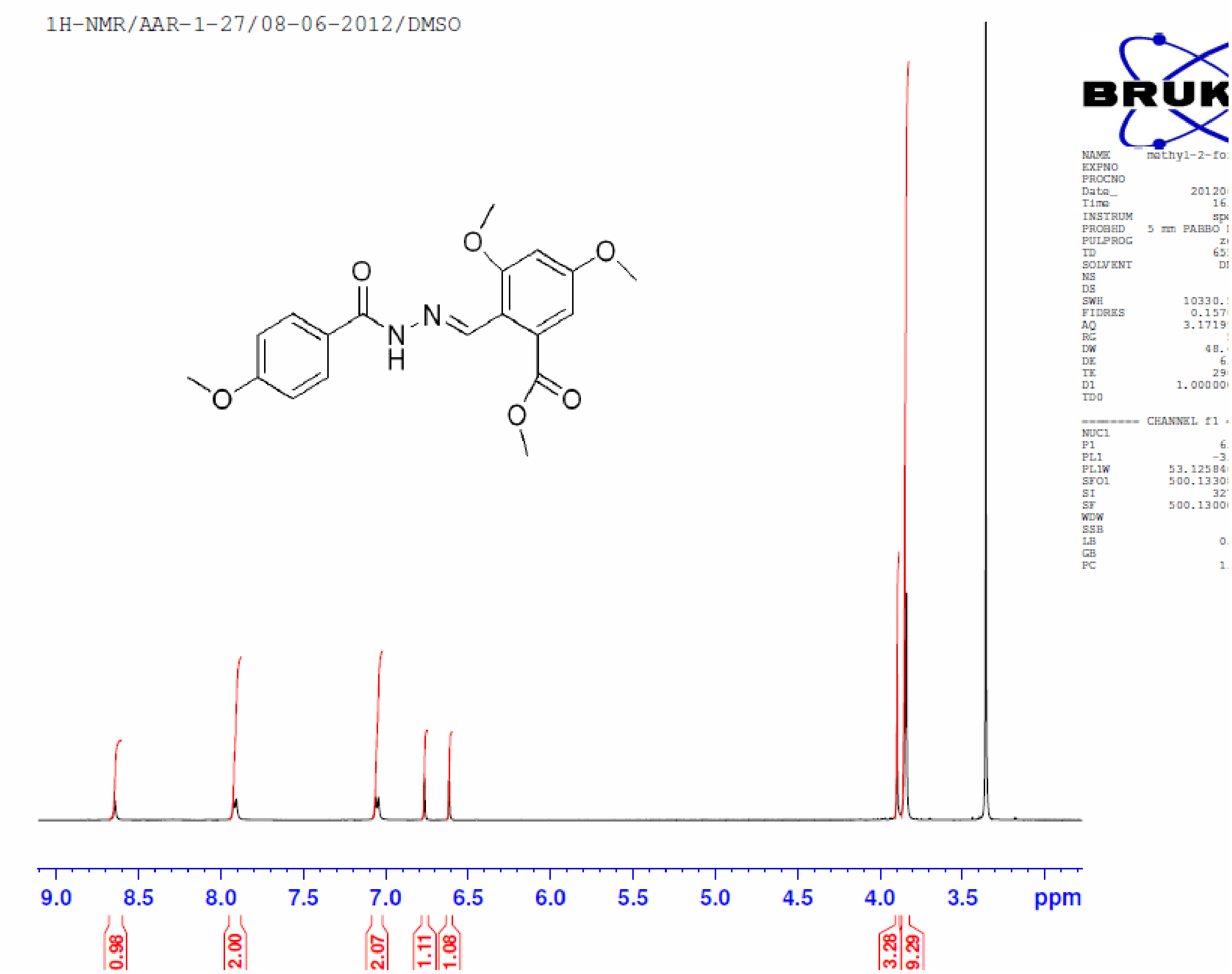

Figure S27. Compound **27**.

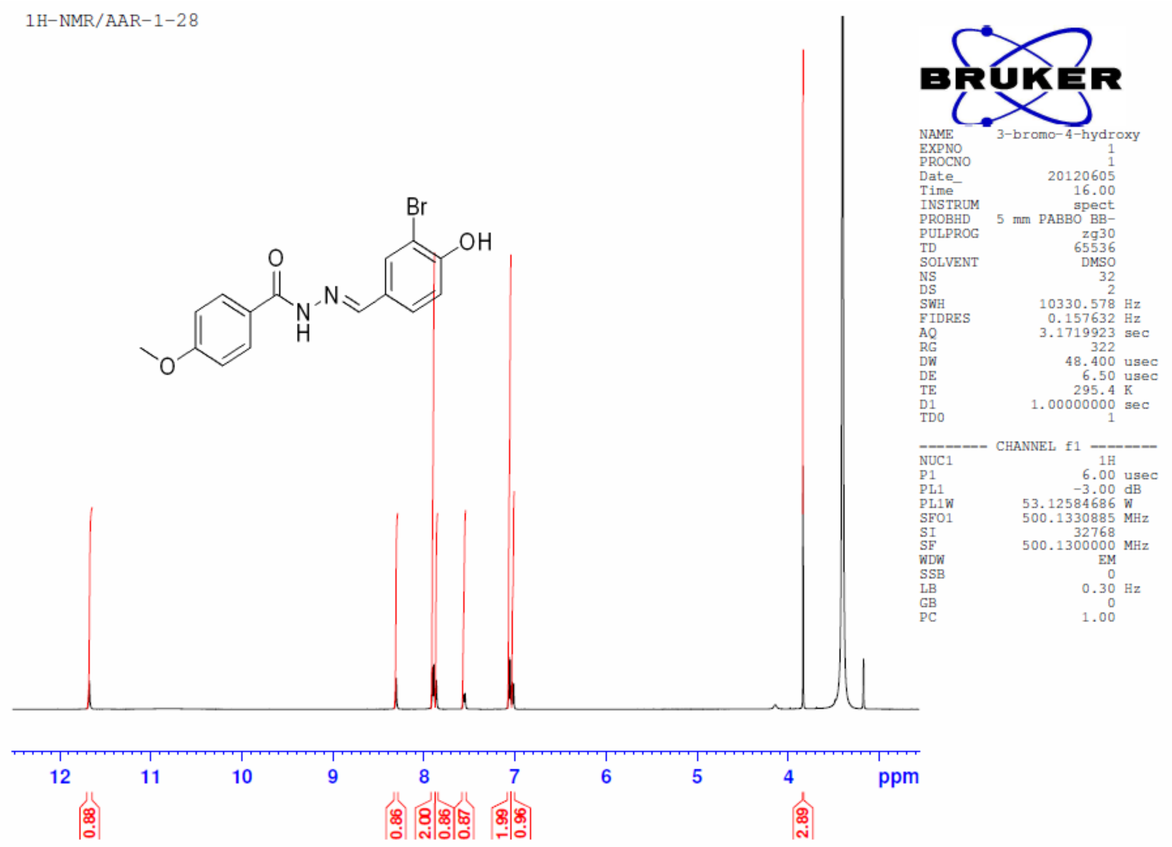

Figure S28. Compound **1**.

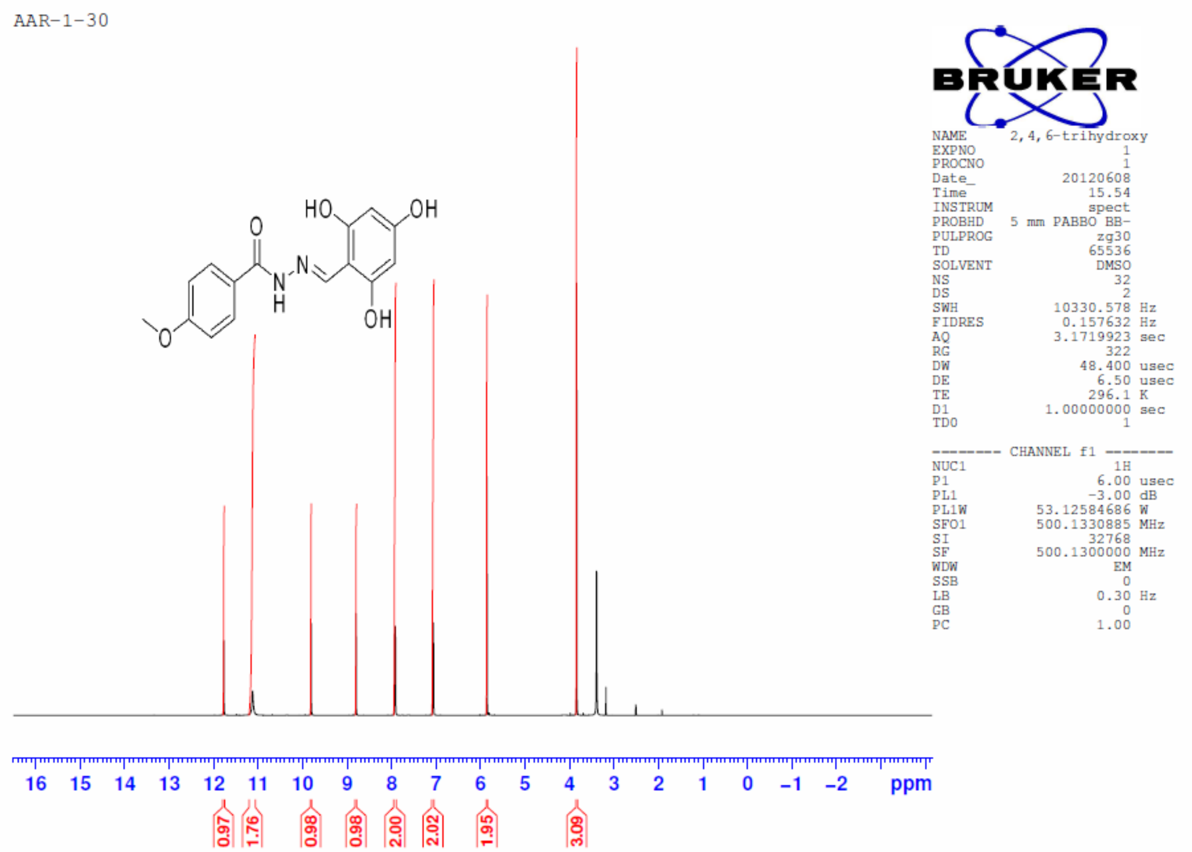

Supplement: Supplementary file 1 [file molecules-19-01286-s001.pdf]
